# Contribution of serum lipids and cholesterol cellular metabolism in lung cancer development and progression

**DOI:** 10.1038/s41598-023-31575-y

**Published:** 2023-04-06

**Authors:** Philipp Hartmann, Denis I. Trufa, Katja Hohenberger, Patrick Tausche, Sonja Trump, Susanne Mittler, Carol I. Geppert, Ralf J. Rieker, Oliver Schieweck, Horia Sirbu, Arndt Hartmann, Susetta Finotto

**Affiliations:** 1 Department of Molecular Pneumology, Friedrich-Alexander-Universität (FAU) Erlangen-Nürnberg, Universitätsklinikum Erlangen, 91052 Erlangen, Germany; 2 Department of Thoracic Surgery, Friedrich-Alexander-Universität (FAU) Erlangen-Nürnberg, Universitätsklinikum Erlangen, 91052 Erlangen, Germany; 3 Institute of Pathology, Friedrich-Alexander-Universität (FAU) Erlangen-Nürnberg, Universitätsklinikum Erlangen, 91052 Erlangen, Germany; 4 Laboratory of Clinic Medicine, Friedrich-Alexander-Universität (FAU) Erlangen-Nürnberg, Universitätsklinikum Erlangen, 91052 Erlangen, Germany; 5 Comprehensive Cancer Center Erlangen-EMN (CCC ER-EMN), Erlangen, Germany; 6 Laboratories of Cellular and Molecular Lung Immunology, Department of Molecular Pneumology, Friedrich-Alexander-Universität Erlangen-Nürnberg, Hartmannstraße 14, 91052 Erlangen, Germany

**Keywords:** Diseases, Medical research, Oncology, Risk factors

## Abstract

Neoplasms of the lungs are the leading cause of cancer incidence and mortality worldwide. Although immunotherapy has increased the overall survival of patients with lung cancer, there is the need to improve this treatment. At this regard, blood lipid levels are thought to be linked to cancer risk and thus a preventive intervention through regulation of the nutrition of patients with lung cancer is gaining much attention. In this study, we therefore asked about the contribution of serum lipids and cholesterol cellular metabolism in lung cancer development and progression. We measured different serum lipids and analyzed cholesterol synthesis enzymes 3-hydroxy-3-methyl-glutaryl-CoA reductase (HMGCR) and acetyl-coenzyme A cholesterol acetyltransferase 1 (ACAT1) as well as the cholesterol cellular export protein ATP-binding cassette (ABC) A-1 mRNA by quantitative PCR (qPCR) in the control and tumoral regions of post-surgery lung tissues to analyze the accumulation of cholesterol in cancer cells in a cohort of patients with lung adenocarcinoma (LUAD). We found that triglycerides in serum directly correlated with the body mass index (BMI) in patients with LUAD. By contrast, we found that high-density lipoprotein (HDL) cholesterol inversely correlated with the BMI, C-reactive protein (CRP) and overall survival and total cholesterol inversely correlated with the tumor diameter, serum CRP and overall survival in these LUAD patients. Functionally, the role of cholesterol is indispensable for the growth and development of normal animal cells where it is tightly regulated. Excess of cellular cholesterol regulated by HMGCR is converted to cholesteryl esters by the enzyme ACAT1 and exported extracellularly by the cholesterol transporter ABCA1. Here we found HMGCR and ACAT1 upregulated and ABCA1 downregulated in the lung’s tumoral region of our LUAD cohort, indicating cholesterol dysregulated cellular export in lung tumor cells.

## Introduction

Neoplasms of the lungs are the leading cause of cancer incidence and mortality worldwide^1^. Although immunotherapy has increased the overall survival of patients with lung cancer, there is the need to improve the success of this treatment^2^. At this regard, a preventive intervention through regulation of the nutrition of the patients with lung cancer is gaining much attention^3^. We recently demonstrated that reducing dietary carbohydrate intake might affect tumor growth, improve current immunotherapy, and target tumor immune escape caused by glucose levels in the tumor microenvironment^4^. In that study, we analyzed a cohort of patients with lung adenocarcinoma (LUAD), a major histologic subtype of non-small-cell lung cancer (NSCLC). As glucose levels in LUAD patients were negatively correlated to postoperative overall survival (OS) rates, glucose-restricted diet emerges as potentially attractive therapeutic concept for patients with lung adenocarcinoma. To continue investigating the connection between nutrition status and the tumor growth, we reasoned that blood lipid levels, as part of the metabolic syndrome, are thought to be linked to cancer risk in general and to lung cancer in particular^5–13^. Furthermore, the serum level of the lipid cholesterol is tightly regulated in normal cells. Tumor cells, in contrast, accumulate cholesterol resulting in a dysregulated cholesterol metabolism in patients with cancer^14,15^. Cholesterol metabolism is important for all cell membrane components and thus it has crucial biological functions in normal cells. In the tumor microenvironment, on the other hand, cholesterol-derived metabolites support cancer progression and suppress immune responses^16^. Usually, excess cellular cholesterol controlled by the cholesterol synthesis enzymes 3-hydroxy-3-methyl-glutaryl-CoAreductase (HMGCR) is converted to cholesteryl esters by the enzyme acetyl-coenzyme A cholesterol acetyltransferase 1 (ACAT1) and then removed from the intracellular spaces via transporters like ATP-binding cassette (ABC) A-1. However, in tumor cells the cholesterol is accumulated into the cells to promote tumor cell growth^17–20^.Thus, the role of lipids and in particular the role of cholesterol homeostasis and export in lung cancer cells is discussed and not clear and needs further investigations^21,22^. It is also accepted that there are lipids with antioxidant property like omega lipids which provide an alternative nutrition avenue activating anti-tumor responses^23^. In this study we investigated the role of serum lipids, cholesterol metabolism and cellular export in lung cancer in a cohort of patients which underwent surgery due to a diagnosis of lung cancer histologically classified as adenocarcinoma (LUAD). This investigation was set up to clarify which lipid components play a major role in lung cancer progression to improve current immunotherapy to this disease^24^.

## Material and methods

This study was approved by appropriate institutions as described below. In addition, all methods were performed in accordance with the relevant guidelines and regulations.

### Human subjects and study population

This study was conducted at the Friedrich-Alexander-University of Erlangen in Germany and was given approval by the ethics review board of the University of Erlangen (Re-No: 56_12B; DRKS-ID: DRKS00005376). In total, one hundred thirty-seven (137) patients with non-small-cell lung cancer (NSCLC) were operated on in the thoracic surgery in Erlangen and gave their consent to participate in this study. Their confidentiality was maintained.

At the time of surgery, the thoracic surgeon took tissue samples from three areas of the surgically removed lung material: From the tumoral area (TU: solid tumor tissue), from the peritumoral area (PT: 2 cm around the tumor) and from the tumor free control area (CTR: at least 5 cm away from the solid tumor). After receiving the samples, the Institute of Pathology at the University Hospital Erlangen confirmed the diagnosis of lung cancer and classified the tumor tissue for its histological subtype, according to the 2015 World Health Organization Classification of Lung Tumors^25^. In addition, the pathologists identified the grading and the TNM stage of the tumor which is based on the Proposals for Revision of the TNM Stage Groupings in the Forthcoming (Eighth) (TNM8) Edition of the TNM Classification for Lung Cancer by the International Association for the Study of Lung Cancer (IASLC), issued in 2017. The abbreviation TNM stands for tumor, nodes and metastasis^26^. All relevant data for this study were provided by the Department of Thoracic Surgery and the Institute of Pathology, University Hospital in Erlangen. The patients’ clinical data are shown in Tables S1, S2 and S3 and are summarized in Table 1.

**Table 1:** Clinical Characteristics of the cohort of patients with NSCLC analyzed in this study.

Characteristics	Mean ± SEM	N
Age—year	65.4 ± 1.041	81
Body weight—kg	78.65 ± 2.023	81
Female sex—no. (%)	50.62	41
Male sex—no. (%)	49.38	40
Body height—m	1.7 ± 0.01050	81
BMI—kg/m^2^	27.15 ± 0.6329	81
Waist circumference—cm	101.6 ± 2.560	27
Tumor diameter—cm	3.477 ± 0.2403	81
Total cholesterol—mg/dl	175.5 ± 5.522	67
HDL cholesterol—mg/dl	45.06 ± 2.084	67
LDL cholesterol—mg/dl	107.9 ± 4.048	67
Triglycerides—mg/dl	125.9 ± 4.723	67
Lipoprotein a—mg/dl	19.71 ± 3.428	67
Smoking—PJ	38.39 ± 3.423	74
Albumin—g/l	41.31 ± 0.5399	60
CRP before—mg/l	10.51 ± 2.226	74
CRP after—mg/dl	113.2 ± 7445	81
Lymphocytes absolute before—× 10^3/ul	1.740 ± 0.07528	80
Lymphocytes before—%	23.79 ± 1.151	80
FEV1—%	83.00 ± 2.404	74
FVC—%	91.66 ± 2.168	68
Glucose—mg/dl	104.8 ± 3.334	61
ACAT1 expression—CTR	0.3483 ± 0.03582	32
ACAT1 expression—TU	0.8955 ± 0.1135	30
HMGCR expression—TU	1.5 ± 1.09	32
HMGCR expression—CTR	0.73 ± 0.37	33
ABCA1 expression—TU	0.68 ± 0.62	30
ABCA1 expression—CTR	1.45 ± 0.86	32

### Control subject cohort

Healthy controls were recruited from two studies currently active at our department. First, the human AZCRA study (investigation of the role of cytokines, chemokines and their receptors in the inflammatory process in asthma patients, n = 19, C501-C547), which was approved by the local ethics committee of the Universitätsklinikum Erlangen at the Friedrich-Alexander-Universität Erlangen-Nürnberg (Re-No. 315_20B). The study is registered in the German Clinical Trial Register (Deutsches Register Klinischer Studien: registration no. DRKS00023843). The other control patients (iK and CN) were recruited from our tumor study (Re-No: 56_12B; DRKS-ID: DRKS00005376). The inclusion criteria for all the control participants are the ability to consent to the study, a medical history without current or former diagnosis of cancer and a minimum age of 18 years. For the AZCRA study further inclusion criteria are a maximum age of 65 years and non-asthmatic and non-atopic condition. The third group of controls (CN, n = 15) was recruited by our collaboration partners in the Thoracic Surgery. This control group included healthy volunteers who donated blood and patients who underwent surgery for benign pathologies (pneumothorax, lung chondroma, etc.). The subjects were at least 18 years old, signed informed consent and did not have history of cancer, chronical diseases, or autoimmune diseases. The characteristics of the 39 control subjects are reported in Tables S4 and S5 and are summarized in Table 2.

**Table 2:** Clinical characteristics of the cohort of control patients analyzed in this study.

Characteristics	Mean ± SEM	N
Age—year	39.38 ± 2.488	37
Female sex—no. (%)	42.11	16
Male sex—no. (%)	57.89	22
Body weight—kg	73.63 ± 2.721	35
Body height—m	1.76 ± 0.01745	35
BMI—kg/m^2^	23.57 ± 0.6269	35
Total cholesterol—mg/dl	186.4 ± 7.249	40
HDL cholesterol—mg/dl	54.25 ± 2.542	40
LDL cholesterol—mg/dl	113.8 ± 5.318	40
Triglycerides—mg/dl	87.23 ± 5.112	40
Lipoprotein a—mg/dl	28.08 ± 5.423	40
Smoking—PJ	14.34 ± 4.155	14

### Lipid measurements

Blood serum samples of our patients with adenocarcinoma were sent to the central laboratory of the University Hospital Erlangen for measurement of the following lipid values: Triglycerides, total cholesterol, high-density lipoprotein (HDL) cholesterol, low-density lipoprotein (LDL) cholesterol and lipoprotein a (Lp (a)). They were all quantitatively determined using an enzymatic color test in Beckman Coulter AU analysis devices except for the determination of Lp (a).

For the triglycerides, a process based on several interrelated enzymatic reactions is used. First, the triglycerides are hydrolyzed to glycerol and three fatty acids by the enzyme lipase. Glycerol is then phosphorylated to glycerol-3-phosphate in a glycerol-kinase dependent manner, which is then in the presence of oxygen and glycerol-oxidase oxidized to hydrogen peroxide (H_2_O_2_) and dihydroxyacetone phosphate. After a series of reactions including 4-Aminophenazone as well as N,N-bis(4-Sulfobutyl)-3,5-Dimethylaniline, H_2_O_2_ produces a chromophore, a blue dye, which can be detected at 660/800 nm. The triglyceride content of the sample can then be determined by measuring its absorbance at 660/800 nm^27,28^. The measurements were accomplished by using the OSR 60118 Triglycerides reagent kit by Coulter Beckman.

To determine the total cholesterol values, we hydrolyzed the cholesterol esters in the sample with the enzyme cholesterol esterase (CHE). As a result, free cholesterol is produced which is then oxidized to cholesten-3-one by the enzyme cholesterol oxidase (CHO). Simultaneously, hydrogen peroxide is formed. Here, too, a chromophore is ultimately formed, after H_2_O_2_ combines with 4-aminoantipyrine and phenol. This reaction is catalyzed by peroxidase (POD). In this case, the chromophore is the red dye quinoneimine which can be measured spectrometrically at 540/600 nm as absorbance increase^29^. For this purpose, the reaction kit OSR 6116 (Beckman Coulter) was used.

To measure the HDL cholesterol value in the blood serum samples, antigen–antibody complexes were formed by adding anti-human-β-lipoprotein antibodies in R1 (reagent 1) of the reagent kit OSR 6187 (Beckman Coulter). The latter bind lipoproteins other than HDL, i.e., LDL, very low-density lipoprotein (VLDL) and chylomicrons. When R2 (reagent 2) is added, the antigen–antibody complexes block further enzymatic reactions, and the HDL cholesterol can be quantified in presence of a chromogenic enzyme system. Here cholesterol esterase and cholesterol oxidase degrade HDL cholesterol to cholest-4-en-3-on, fatty acids and H_2_O_2_ whereat the peroxide is used to form a blue chromophore in a peroxidase dependent reaction.

Lastly, we will take a closer look at how the LDL cholesterol was measured: By reaction with cholesterol esterase (CHE) and cholesterol oxidase (CHO), all lipoproteins in R1 (OSR 6183, Beckman Coulter): LDL, VLDL and chylomicrons except for LDL are degraded and formed hydrogen peroxide is then decomposed by the enzyme catalase. The LDL in R1 is protected from these enzymatic reactions by a protective agent. To finally quantify LDL with the CHO/PAP-method, R2 is added. In this way the protective agent is released from the LDL and catalase is deactivated by sodium azide. Cholesterol esterase as well as cholesterol oxidase are deployed whereby LDL cholesterol is degraded to cholest-4-en-3-on, fatty acids and hydrogen peroxide. Subsequently a peroxidase catalyzes the reaction of peroxide, aminophenazone and phenol, forming a blue dye^30^.

The determination of Lp (a) was performed by use of the analysis device Integra 400 (Roche) due to a particle-enhanced immunoturbidimetric assay (LPA2, Tina-quant-Lipoprotein (a) Gen.2, Roche), where human Lp (a) agglutinates with latex particles coated with anti-lipoprotein (a) antibodies. Following precipitation is determined turbidimetrically at 659 nm^31^.

### Quantitative Real-Time PCR (qPCR)

Frozen lung tissue samples were homogenized using Precellys Lysing Kits (Bertin Technologies, Cat# P000918-LYSK0-A) and the homogenizer Minilys (Bertin Technologies) as described in the manufacturer’s protocol. Total RNA was extracted from homogenized samples using Qiazol Lysis® Reagent (QIAGEN, Cat#79306) according to the manufacturer's instructions. Subsequently, 1 µg of RNA was reverse transcribed into copy DNA (cDNA) via the RevertAid™ First Strand cDNA Synthesis Kit (ThermoFisher Scientific, Cat#K1622) according to the manufacturer's protocol. Each qPCR reaction mix contained 5 ng of cDNA in a total volume of 20 µl and was performed using the iTaq Universal SYBR Green Supermix (Bio-Rad Laboratories, Cat# 1725124). Primers for qPCR analysis were purchased from Eurofins-Genomics Germany and sequences are shown in Supplementary Table S6. Reactions (50 cycles, initial activation 98 °C, 2 min, denaturation 95 °C, 5 min, hybridization/elongation 60 °C, 10 min) were performed using the CFX-96 Real-Time PCR Detection System (BIO-RAD, Munich, Germany) and analyzed via the CFX Manager Software (BIO-RAD, Munich, Germany). The relative expression level of specific transcripts was calculated using the relative quantification ΔΔCq method by normalization to the housekeeping-gene 60S ribosomal protein L30 (RPL30) or Hypoxanthine–guanine phosphoribosyltransferase (HPRT).

### Statistical analysis

The collected data were imported in column statistics and were then analyzed. We evaluated differences by using GraphPad PRISM 8 for significance (*P = 0.05; **P = 0.01; ***P = 0.001; ****P = 0.0001). For two dependent columns the paired t-test was performed. To examine correlations, we imported data in XY tables and diagrammed them with linear regression curve. Survival data are shown in Kaplan–Meier survival curves.

Data are given as mean values ± SEM. The graphs were created with GraphPad Prism 8.

## Results

### Clinical characteristics of the cohort of patients with NSCLC (LUAD) analyzed in this study

**Figure 1:**
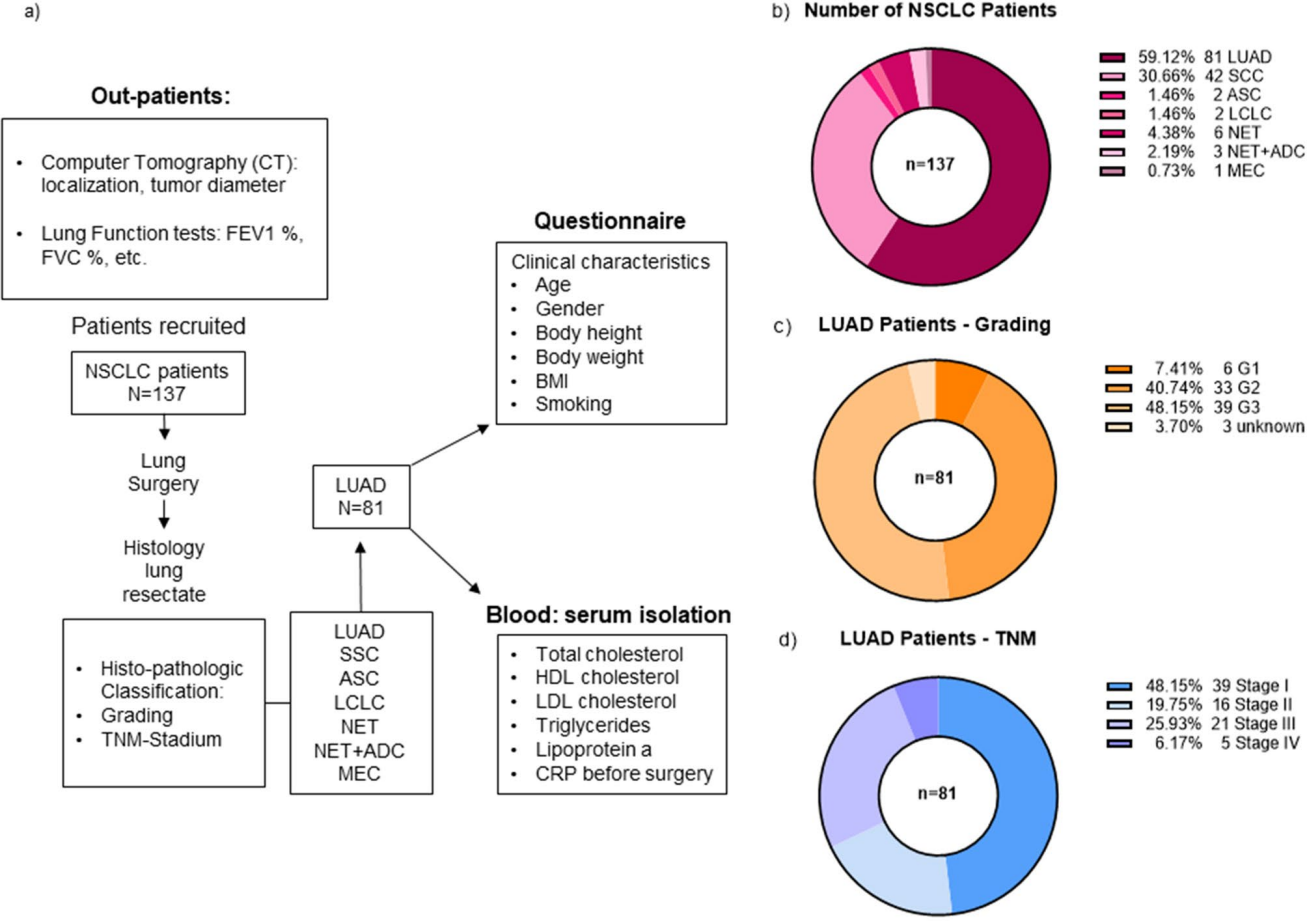
Clinical characteristics of the cohort of patients with NSCLC (LUAD) analyzed in this study. (**a**) Workflow of the study (*LUAD* adenocarcinoma, *SCC* squamous cell carcinoma, *ASC* adenosquamous carcinoma, *LCLC* large cell lung carcinoma, *NET* neuroendocrine tumor, *NET + ADC* adeno-neuroendocrine carcinoma, *MEC* mucoepidermoid carcinoma). (**b**) Diagram of distribution of our patients’ cohort depending on the histological classification (LUAD: n = 81, SCC: n = 42, ASC: n = 2, LCLC: n = 2, NET: n = 6, NET + ADC: n = 3, MEC: n = 1). (**c**) Diagram of distribution of our patients’ cohort depending on the grading (G1: n = 6, G2: n = 33, G3: n = 39, unknown: n = 3). (**d**) Diagram of distribution of our patients’ cohort depending on the TNM classification (Stage I: n = 39, Stage II: n = 16, Stage III: n = 21, Stage IV: n = 5).

The patients were enrolled in this study by the Thoracic Surgery Department at the UKER (University Hospital Erlangen). At the time of analysis, all patients underwent surgery with first curative intent and the majority of them were in early stages of lung cancer with only four of them having metastasis. After the initial examination in the outpatient clinic, a Computer Tomography (CT) imaging at the radiology Department with a diagnosis of non-small-cell lung cancer (NSCLC) was done. The CT imaging helped the thoracic surgeon to exactly localize the tumor as well as its size. In addition, pulmonary function tests were required to assess relevant values like the forced expiratory volume (FEV1) and the forced vital capacity (FVC). They were performed at the MED I Clinic at UKER (Fig. 1a).

In total 137 patients with non-small-cell lung cancer (NSCLC) were recruited into this study. All of them underwent lung surgery. The surgically removed tumor tissue was then sent to the Institute of Pathology at the University Hospital Erlangen for further histological classification. Apart from identifying the grading and the TNM stage of the tumor, the histological subtype was determined. In this study, we focused on the patients with adenocarcinoma (LUAD). In addition to complete questionnaires providing information about clinical characteristics like age, gender and body height, blood was taken from all patients for measuring total cholesterol, HDL cholesterol, LDL cholesterol, triglycerides, and lipoprotein a (Fig. 1a).

Tables S1, S2 and S3 provide an overview of all the main characteristics of the LUAD patients that were analyzed in this study. The mean values of these characteristics with the standard error of the mean (SEM) can be viewed in the Table 1.

Out of the 137 patients with NSCLC, 81 patients were classified for lung adenocarcinoma (59.12%) (LUAD), followed by 42 participants with lung squamous-cell carcinoma (SCC). The remaining ten percent are made up of rarer subtypes like the neuroendocrine tumor (NET) with 4.38% and the adenosquamous carcinoma (ASC) with 1.46% (Fig. 1b).

When looking at the grading of the patients with LUAD the majority shows moderately (G2, 40.74%) or poorly (G3, 48.15%) differentiated tumor tissue. Only 7.41% can be assigned to G1 (Fig. 1c).

Regarding the TNM classification almost half of the patients show tumors at Stage I (48.15%). 25.99% of patients show Stage III tumors while 19.75% show tumors at Stage II. Only 6.17% of the patients have Stage IV tumor (Fig. 1d).

### Tumor classification of LUAD patients in dependence of the BMI

**Figure 2:**
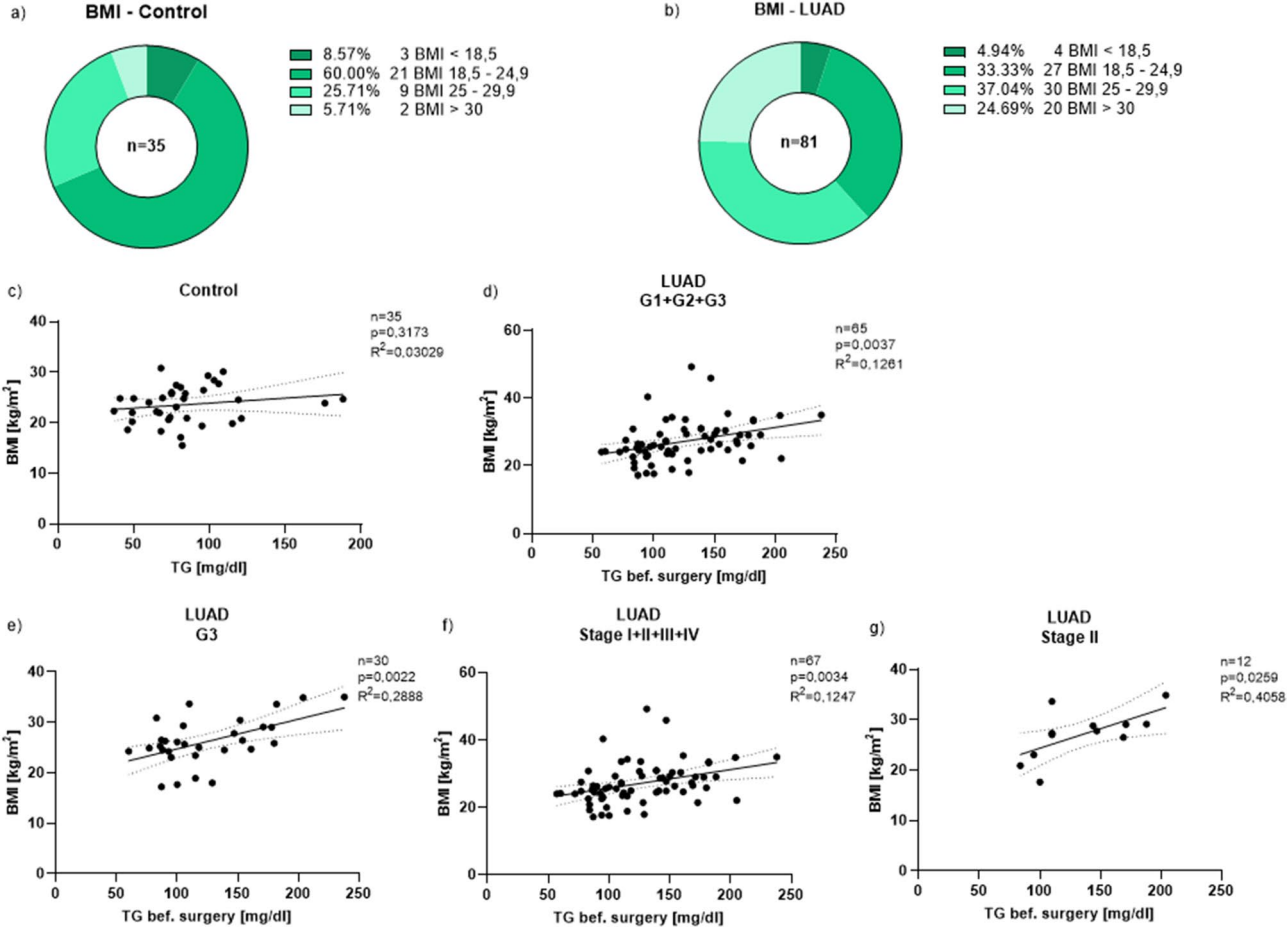
Pre-surgical triglyceride values directly correlate with the BMI in dependence of the grading/the TNM classification. (**a**) Diagram of distribution of our control patients’ cohort depending on the BMI (BMI < 18.5 (underweight): n = 3; BMI 18.5–24.9 (normal weight): n = 21; BMI 25–29.9 (overweight): n = 9; BMI > / = 30 (obese): n = 2). (**b**) Diagram of distribution of our LUAD patients’ cohort depending on the BMI (BMI < 18.5 (underweight): n = 4; BMI 18.5–24.9 (normal weight): n = 27; BMI 25–29.9 (overweight): n = 30; BMI > / = 30 (obese): n = 20). (**c**) Direct correlation between the triglyceride values and the BMI of all control patients (n = 35, p = 0.3173, R^2^ = 0.03029). (**d**) Direct correlation between the triglyceride values before surgery and the BMI of all LUAD patients (G1 + G2 + G3) in dependence of the grading (n = 65, p = 0.0037, R^2^ = 0.1261). (**e**) Direct correlation between the triglyceride values before surgery and the BMI of all LUAD patients with G3 in dependence of the grading (n = 30, p = 0.0022, R^2^ = 0.2888). (**f**) Direct correlation between the triglyceride values before surgery and the BMI of all LUAD patients (Stage I + II + III + IV) in dependence of the TNM classification (n = 67, p = 0.0034, R^2^ = 0.1247). (**g**) Direct correlation between the triglyceride values before surgery and the BMI of all LUAD patients with Stage II in dependence of the TNM classification (n = 12, p = 0.0259, R^2^ = 0.4058). Correlations are shown by using simple linear regression.

In order to understand the contribution of nutrition status on the lung tumor and to get a better understanding of the patients’ nutrition status we looked at their body mass index (BMI) which can be used as an indicator for body mass and food intake. For this purpose, we divided our cohorts of patients into four groups: participants with a BMI smaller than 18.5 kg/m^2^ (underweight), with a BMI between 18.5 and 24.9 kg/m^2^ (normal weight), with a BMI between 25 to 29.9 kg/m^2^ (overweight) and with a BMI greater than or equal to 30 kg/m^2^ (obese)^32^. For a direct comparison to the tumor group, we also evaluated the BMI of the control patients (Fig. 2a,b). In the control group, 60% of the participants had normal weight, only 31.42% were overweight/obese (Fig. 2a). Most patients with LUAD (61.73%), on the other hand, showed an above-average BMI. Only a small percentage was underweight (4.94%) (Fig. 2b).

Next, we wanted to find out how high the patients’ BMI was, depending on the grading of the LUAD. The average BMI of the tumor patients (G1: 29.8 kg/m^2^; G2: 27.4 kg/m^2^; G3: 26.7 kg/m^2^) is comparable to the BMI of the control patients (Control: 23.6 kg/m^2^) (Supplementary Fig. S1a). Patients with tumor tissue classified as G1 have the highest mean BMI, followed by G2 and then G3. The average BMI of all these groups was higher than 25 kg/m^2^ whereas in the control group it was smaller than 25 kg/m^2^.

The situation is similar when looking at the TNM classification: The average BMI in all four stages (Stage I: 28.4 kg/m^2^; Stage II: 26.8 kg/m^2^; Stage III: 25.8 kg/m^2^; Stage IV: 23.9 kg/m^2^) was by trend higher than in the control group (Control: 23.6 kg/m^2^), but not statistically different (Supplementary Fig. S1b).

We next wanted to examine if the blood glucose levels of tumor patients have influence on their BMI. Therefore, we correlated these two parameters and discovered that they were not dependent on each other (Supplementary Fig. S1c).

### Pre-surgical triglyceride values directly correlate with the BMI in dependence of the grading/the TNM classification

Next, we wanted to investigate whether the patients’ lipid values and their BMI influence each other, especially in dependence of the grading and the TNM classification. Therefore, we first took a closer look at the triglyceride values that were measured before surgery depending on the grading. It became apparent that the mean triglyceride value of the tumor patients in all three subgroups (G1, G2 and G3) was still within the normal reference range of under 150 mg/dl (G1: 146.2 mg/dl; G2: 121.4 mg/dl; G3: 125 mg/dl) (Supplementary Fig. S2a). However, it was higher than in the control patients’ group (Control: 87.2 mg/dl). Looking at the higher triglyceride value of each patient separately, there are way more tumor patients (n = 16) with triglyceride values above 150 mg/dl in comparison to the control patients (n = 2) (Supplementary Fig. S2a,b).

To assess whether triglyceride values in the blood are influenced by the BMI of patients, we then correlated the triglyceride values of all control subjects and patients with G1, G2 and G3 tumor tissue with the BMI and found a significant correlation between these two parameters in patients with LUAD but not in control subjects (Fig. 2c,d). Looking at the grading subgroups individually, especially the LUAD patients with G3 tissue showed a direct significant correlation between the pre-surgical triglyceride values and their BMI (Fig. 2e). After having taken the tumor grading into consideration, we then looked at the TNM classification and how the triglyceride values are dependent on it. We observed that similar to Supplementary Fig. S2a the patients’ triglyceride values were in average higher than in the control group (Stage I: 143.4 mg/dl; Stage II: 136 mg/dl; Stage III: 102.8 mg/dl; Stage IV: 129.6 mg/dl; control group: 87.2 mg/dl) (Supplementary Fig. S2b). When correlating the triglycerides of all tumor patients (Stage I, II, III and IV) with the BMI there was a direct significant correlation again (Fig. 2f). For patients with Stage II tumor, we could also find a direct significant correlation (Fig. 2g). In conclusion, all these correlations showed a direct dependence of the BMI of patients with lung cancer with rising triglyceride levels (Fig. 2d–g).

### Pre-surgical HDL cholesterol values inversely correlate with the BMI in dependence of the grading/the TNM classification

To further investigate the role of lipids in the development of lung cancer we analyzed the HDL cholesterol of all participants, first depending on the grading. Here we found that, the tumor patients’ HDL cholesterol values which were measured in blood before surgery were on average still within the healthy reference range of 40 to 60 mg/dl (G1: 48.8 mg/dl, G2: 41.1 mg/dl, G3: 48 mg/dl). In comparison to the control group (Control: 54.3 mg/dl) however, their mean HDL cholesterol value was lower (Supplementary Fig. S2c).

**Figure 3:**
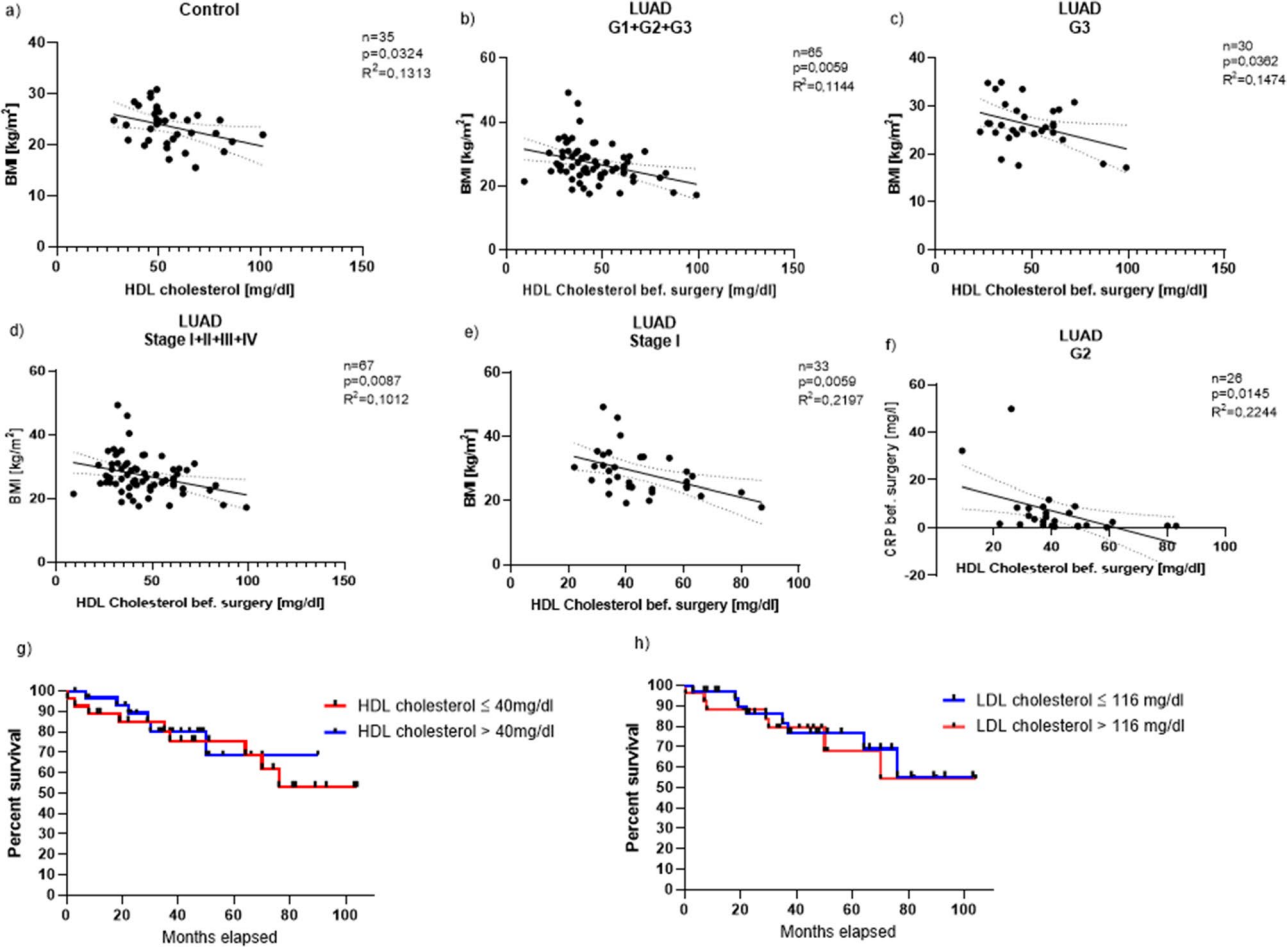
Pre-surgical HDL cholesterol values inversely correlate with the BMI in dependence of the grading/the TNM classification. (**a**) Direct correlation between the HDL cholesterol values and the BMI of all control patients (n = 35, p = 0.0324, R^2^ = 0.1313). (**b**) Direct correlation between the HDL cholesterol values before surgery and the BMI of all LUAD patients (G1 + G2 + G3) in dependence of the grading (n = 65, p = 0.0059, R^2^ = 0.1144). (**c**) Direct correlation between the HDL cholesterol values before surgery and the BMI of all LUAD patients with G3 in dependence of the grading (n = 30, p = 0.0362, R^2^ = 0.1474). (**d**) Direct correlation between the HDL cholesterol values before surgery and the BMI of all LUAD patients (Stage I + II + III + IV) in dependence of the TNM classification (n = 67, p = 0.0087, R^2^ = 0.1012). (**e**) Direct correlation between the HDL cholesterol values before surgery and the BMI of all LUAD patients with Stage I in dependence of the TNM classification (n = 33, p = 0.0059, R^2^ = 0.2197). (**f**) Direct correlation between the HDL cholesterol values before surgery and the CRP values before surgery of all LUAD patients with G2 in dependence of the grading (n = 26, p = 0.0145, R^2^ = 0.2244). (**g**) Kaplan–Meier curve depending on HDL-cholesterol level in the serum before surgery (red line: HDL cholesterol ≤ 40 mg/dl: n = 30; blue line: HDL cholesterol > 40 mg/dl: n = 37). (**h**) Kaplan–Meier curve depending on LDL-cholesterol level in the serum before surgery (blue line: LDL cholesterol ≤ 116 mg/dl: n = 39; red line: LDL cholesterol > 116 mg/dl: n = 28) . Correlations are shown by using simple linear regression.

We then correlated the HDL cholesterol in controls (Fig. 3a) and in the tumor group (G1, G2, G3) (Fig. 3b) with the BMI. We observed a significant inverse correlation between the two in the tumor group and in the control group (Fig. 3a, b). We then also performed this analysis for each individual subgroup, i.e., for patients with G1, G2 and G3 tumor tissue. In patients with G3 cancer, we confirmed a significant inverse correlation between their BMI and the blood HDL cholesterol value (Fig. 3c). We also wanted to know how the HDL cholesterol relates to the TNM classification. Similar to the grading, the tumor patients’ mean HDL cholesterol values of all stages were still within the normal range except for patients with Stage IV tumor (Stage I: 45 mg/dl; Stage II: 42.9 mg/dl; Stage III: 48.4 mg/dl). Here the mean HDL cholesterol was 37 mg/dl. Overall, the HDL cholesterol values of all four tumor subgroups were in average lower than in the control group (Control: 54.2 mg/dl) (Supplementary Fig. S2d).

Again, we correlated the HDL cholesterol values of all patients (Stage I, II, III and IV) with their BMI and discovered that there was a significant inverse correlation between the two (Fig. 3d). After that we analyzed each TNM subgroup separately again and correlated HDL cholesterol with the BMI which resulted in a significant inverse correlation in patients with Stage I (Fig. 3e) as well as with Stage IV tumor (Supplementary Fig. S4a). Altogether, the BMI level decreases the higher the value of HDL cholesterol (Fig. 3b–e; Fig. S4a) in our cohort of patients with LUAD. We next analyzed lipoprotein a in our cancer patients and found a significant inverse correlation between lipoprotein a^33^ and the BMI in patients with advanced stadium of LUAD (Stage IV) (Supplementary Fig. S4b). We were also interested in how the CRP value measured before surgery was related to the HDL cholesterol values of the LUAD patients. When correlating these two values, an inverse correlation with a significance of p = 0.0145 could be found (Fig. 3f). Finally, we analyzed the survival of the patients subdividing those with low and high serum HDL cholesterol and found that patients with higher HDL cholesterol had increased overall survival (Fig. 3g). By contrast, by analyzing the patients in low and high LDL cholesterol no difference in overall survival was observed (Fig. 3h).

### Pre-surgical total cholesterol values inversely correlate with the tumor diameter in dependence of the grading/the TNM classification

**Figure 4:**
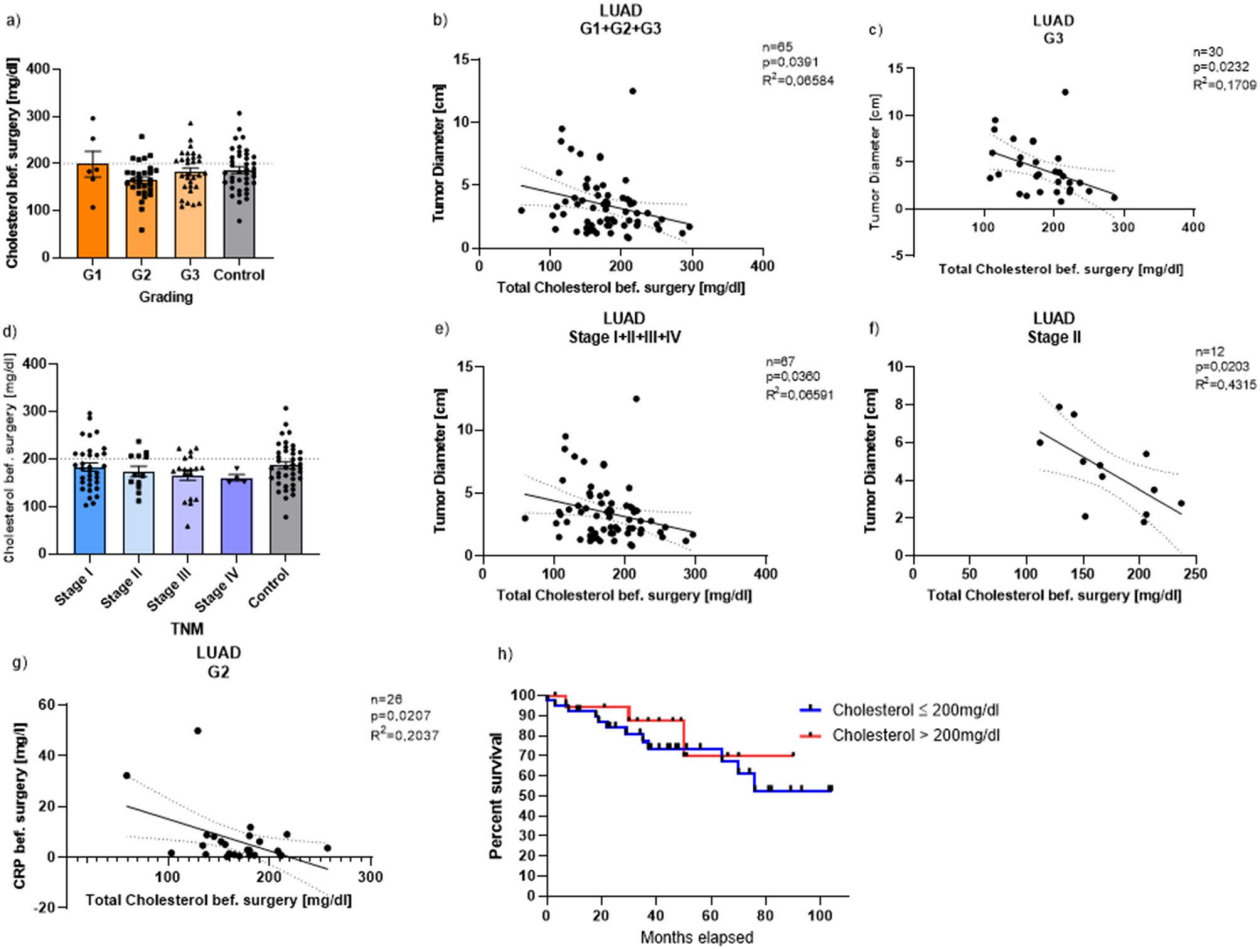
Pre-surgical total cholesterol values inversely correlate with the tumor diameter in dependence of the grading/the TNM classification. (**a**) Total cholesterol values before surgery in dependence of the grading (Mean: G1: 198.8; G2: 164.4; G3: 182.7; control group: 186.4). (**b**) Direct correlation between the total cholesterol values before surgery and the tumor diameter of all LUAD patients (G1 + G2 + G3) in dependence of the grading (n = 65, p = 0.0391, R^2^ = 0.06584). (**c**) Direct correlation between the total cholesterol values before surgery and the tumor diameter of all LUAD patients with G3 in dependence of the grading (n = 30, p = 0.0232, R^2^ = 0.1709). (**d**) Total cholesterol values before surgery in dependence of the TNM classification (Mean: Stage I: 183.2; Stage II: 173.6; Stage III: 165.9; Stage IV: 160.3; Control group: 186.4). (**e**) Direct correlation between the total cholesterol values before surgery and the tumor diameter of all LUAD patients (Stage I + II + III + IV) in dependence of the TNM classification (n = 67, p = 0.0360, R^2^ = 0.06591). (**f**) Direct correlation between the total cholesterol values before surgery and the tumor diameter of all LUAD patients with Stage II in dependence of the TNM classification (n = 12, p = 0.0203, R^2^ = 0.4315). (**g**) Direct correlation between the total cholesterol values before surgery and the CRP values before surgery of all LUAD patients with G2 in dependence of the grading (n = 26, p = 0.0207, R^2^ = 0.2037). (**h**) Kaplan–Meier curve depending on total cholesterol level in the serum before surgery (blue line: total cholesterol ≤ 200 mg/dl: n = 45; red line: total cholesterol > 200 mg/dl: n = 22). Correlations are shown by using simple linear regression. Data are shown as mean values ± s.e.m.

In addition to the triglycerides and the HDL cholesterol, the total cholesterol plays an important role in the pathogenesis of adenocarcinoma, too. Therefore, we first looked at the patients’ total cholesterol values measured before surgery in dependence of the grading and found that the tumor patients’ mean total cholesterol values of all three subgroups (G1, G2 and G3) as well as the mean cholesterol values of the control group were within the reference range of under 200 mg/dl (G1: 198.8 mg/dl, G2: 164.4 mg/dl, G3: 182.7 mg/dl, Control: 186.4 mg/dl). However, there were several patients in all four groups with values above 200 mg/dl (Fig. 4a).

Since we wondered whether the lipid levels influence the tumor diameter, we correlated the two values and found a significant inverse correlation between the tumor diameter and the total cholesterol of all tumor patients (G1, G2 and G3). With increasing total serum cholesterol, the tumor diameter decreased (Fig. 4b). We then performed this analysis again for each subgroup individually and there was a significant inverse correlation between the two values in patients of the third subgroup (G3). The tumor diameter decreased the higher the total cholesterol was (Fig. 4c).

We then looked at the relation of total cholesterol and the TNM classification: The mean total cholesterol in all four TNM subgroups was similarly high and was still within the normal range (Stage I: 183.2 mg/dl; Stage II: 173.6 mg/dl; Stage III: 165.9 mg/dl; Stage IV: 160.3 mg/dl). The same applies to the control group (Control: 186.4 mg/dl). Here, some patients also had significantly elevated total cholesterol values which is noticed when looking at them separately (Fig. 4d). When we correlated the total cholesterol values of all tumor patients (Stage I, II, III, IV) with the tumor diameter, an inverse correlation with a significance of p = 0.0360 resulted (Fig. 4e). Patients with Stage II tumor also showed a significant inverse correlation between these two values: the tumor size was negatively dependent on the total cholesterol (Fig. 4f). Lastly, we took a closer look at the C-reactive protein (CRP) measured before surgery and correlated it with the total cholesterol values of our tumor patients. Here, we also discovered a significant inverse correlation for LUAD patients with G2 cancer (Fig. 4g). Consistently patients with higher total cholesterol level had increased overall survival (Fig. 4h).

### Increased cholesterol biosynthesis pathway in lung cancer cells caused by the overexpression of HMGCR and ACAT1 in the tumoral lung region of patients with LUAD

To understand why serum total cholesterol and tumor diameter were inversely correlated, we took a closer look at the cholesterol biosynthesis pathway in the lung. We reasoned that in the cholesterol biosynthesis at first, two acetyl CoA molecules condensate into acetoacetyl CoA which is catalyzed by the enzyme thiolase. Acetoacetyl CoA is then converted to 3-hydroxy-3-methylglutaryl coenzyme A (HMG-CoA) by HMG-CoA synthase followed by the reduction of HMG-CoA to Mevalonate. The enzyme responsible for this step is called HMG-CoA reductase (HMGCR). After several more successive reactions the end product cholesterol is formed^34^.

**Figure 5:**
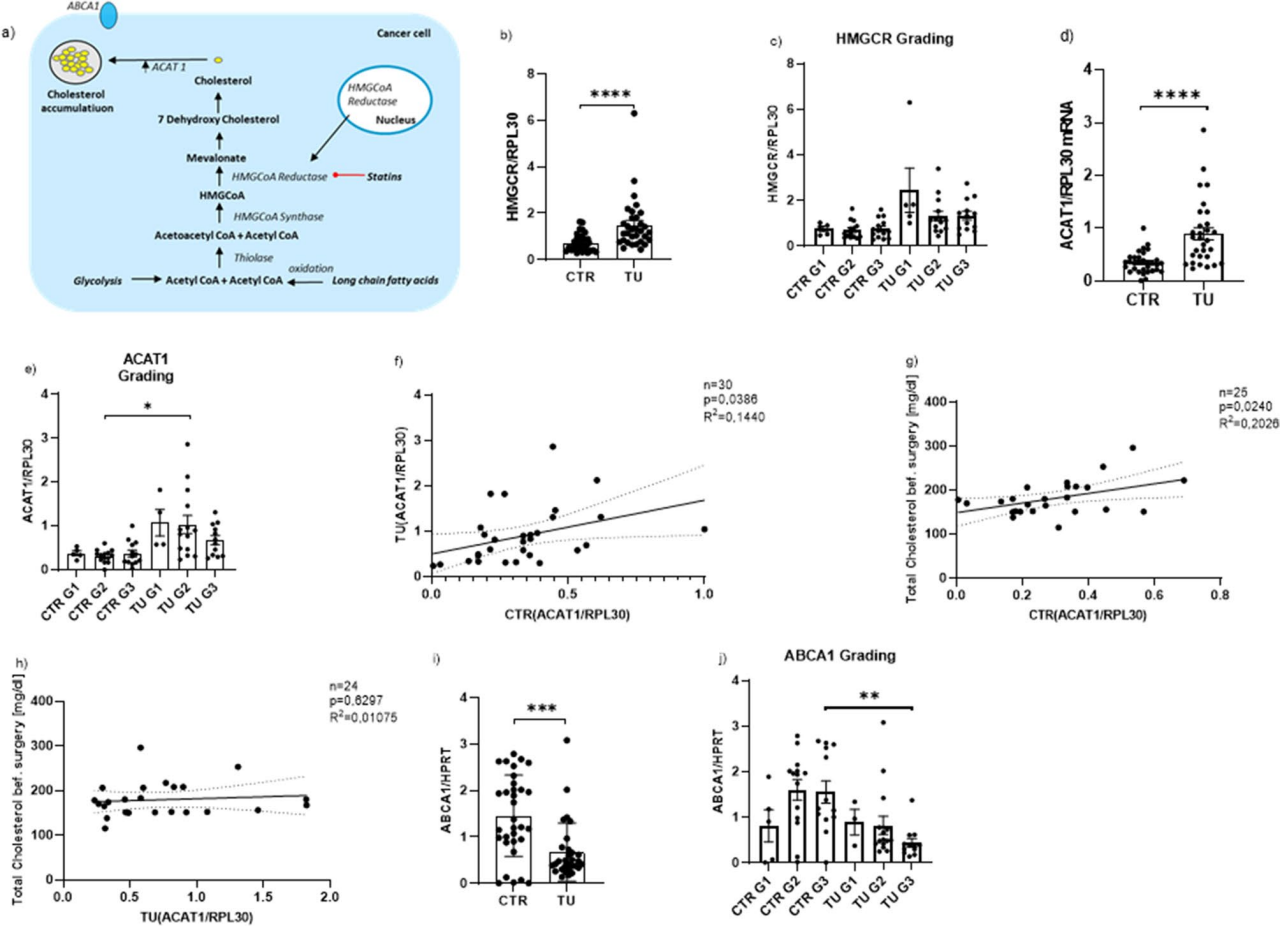
Increased cholesterol biosynthesis pathway in lung cancer cells caused by the overexpression of HMGCR and ACAT1 in the tumoral lung region of patients with LUAD and qPCR analysis of relative ACAT1/RPL30, HMGCR/RPL30 and ABCA1/HPRT mRNA expression level in patients with LUAD. (**a**) Cholesterol biosynthesis pathway and the accumulation of cholesterol caused by an overexpression of ACAT1 in cancer cells. (**b**) qPCR analysis of relative HMGCR/RPL30 mRNA expression level, measured in the control region and tumoral region of patients with LUAD (CTR:n = 33; TU: n = 32). (**c**) qPCR analysis of HMGCR/RPL30 mRNA expression level subdivided into tumor gradings (CTR G1: n = 5; CTR G2: n = 14; CTR G3: n = 14; TU G1: n = 5; TU G2: n = 14; TU G3: n = 13). (**d**) qPCR analysis of relative ACAT1/RPL30 mRNA expression level in patients with LUAD: control region (CTR) and tumoral region (TU). (CTR n = 32; TU n = 30, p (CTR vs. TU) =  < 0.0001). (**e**) qPCR analysis of ACAT1/RPL30 mRNA expression level subdivided into tumor gradings (CTR G1:n = 5; CTR G2: n = 14; CTR G3: n = 13; TU G1: n = 4; TU G2: n = 14; TU G3: n = 12, p (CTR G2 vs. TU G2) = 0.0125). (**f**) qPCR analysis of relative ACAT1/RPL30 mRNA expression level measured in the control region correlated with relative ACAT1/RPL30 mRNA expression level measured in tumoral region of patients with LUAD (n = 30, p = 0.1440, R^2^ = 0.0386). (**g**) qPCR analysis of relative ACAT1/RPL30 mRNA expression level measured in the control region (CTR) correlated with total cholesterol values of LUAD patients (n = 25, p = 0.240, R^2^ = 0.2026). (**h**) qPCR analysis of relative ACAT1/RPL30 mRNA expression level measured in the tumoral region (TU) correlated with total cholesterol values of LUAD patients (n = 24, p = 0.6297, R^2^ = 0.01075). (**i**) qPCR analysis of relative ABCA1/HPRT mRNA expression level, measured in the control region and tumoral region of patients with LUAD (CTR:n = 33; TU: n = 32). (**j**) qPCR analysis of ABCA1/HPRT mRNA expression level subdivided into tumor gradings (CTR G1:n = 5; CTR G2: n = 14; CTR G3: n = 13; TU G1: n = 3; TU G2: n = 14; TU G3: n = 12, p (CTR G3 vs. TU G3) = 0.0085). Correlations are shown by using simple linear regression. Paired t-test was performed for Figure b,d and i; Kruskal Wallis test was performed for Figure e and j. Data are shown as mean values ± s.e.m.; *P < 0.05; **P < 0.01***P < 0.001 ****P < 0.0001.

Usually, the presence of cholesterol regulates its metabolism itself in normal cells. If the intracellular cholesterol level is too high for example the cholesterol biosynthesis is downregulated, and cholesterol efflux transporters are increasingly expressed^35^. In cancer cells, however, this mechanism is disturbed. Instead, there is an overexpression of HMGCR reductase and the presence of intracellular cholesterol leads to an increased expression of ACAT1, an enzyme which catalyzes the conversion of cholesterol to cholesterol ester^35,36^. Due to the overexpression of ACAT1 the cholesterol accumulates more and more in the cancer cells^35^ (Fig. 5a).

To find out how ACAT1 is involved in the development of lung cancer we isolated RNA from the control and tumoral regions of our LUAD patients’ resectated lung tissue. After RNA isolation, cDNA was synthetized. Subsequently, quantitative PCR was performed to measure HMGCR and ACAT1 mRNA expression in the lung tissue of our cancer patients. This analysis showed that both the HMGCR (Fig. 5b,c) as well as ACAT1 (Fig. 5d,e) mRNA in relation to RPL30 mRNA expression level in the tumoral region were significantly higher than in the control region of the lung . Considering the grading of lung cancer and how the HMGCR and ACAT1 mRNA expression behaved in dependence of the single grading subgroups we found that ACAT1 expression showed a significant increase in the tumoral region of patients with G1 and G2 lung cancer (Fig. 5e). As far as the TNM classification is concerned there was also a higher expression of HMGCR and ACAT1 mRNA in the tumoral region in comparison to the control region, especially in Stage II for both genes and Stage IV tumor tissue for ACAT1 (Supplementary Fig. S5a, b). Furthermore, to investigate if the ACAT1 mRNA expression in the tumoral and in the control region influence each other, we correlated the two parameters and found a significantly positive correlation (Fig. 5f).

### Direct correlation between the ACAT1/RPL30 and the total serum cholesterol values in the control (CTR) but not in the tumoral lung region in our cohort of subjects with NSCLC before surgery

Since the enzyme ACAT1 leads to an increased accumulation of total cholesterol in cancer cells^35^, we next analyzed the ACAT1 mRNA expression levels in the control as well as in the tumoral region and found a direct significant correlation between the total cholesterol in the blood and the ACAT1 mRNA expression in the control region of the lung tissue resectates (Fig. 5g). The ACAT1 mRNA expression increased, the higher the total blood cholesterol level was. In the tumor region, however, this analysis did not show any correlation (Fig. 5h).

### Decreased expression of the cholesterol transporter ABCA1 mRNA in the tumoral lung region of patients with LUAD

**Figure 6:**
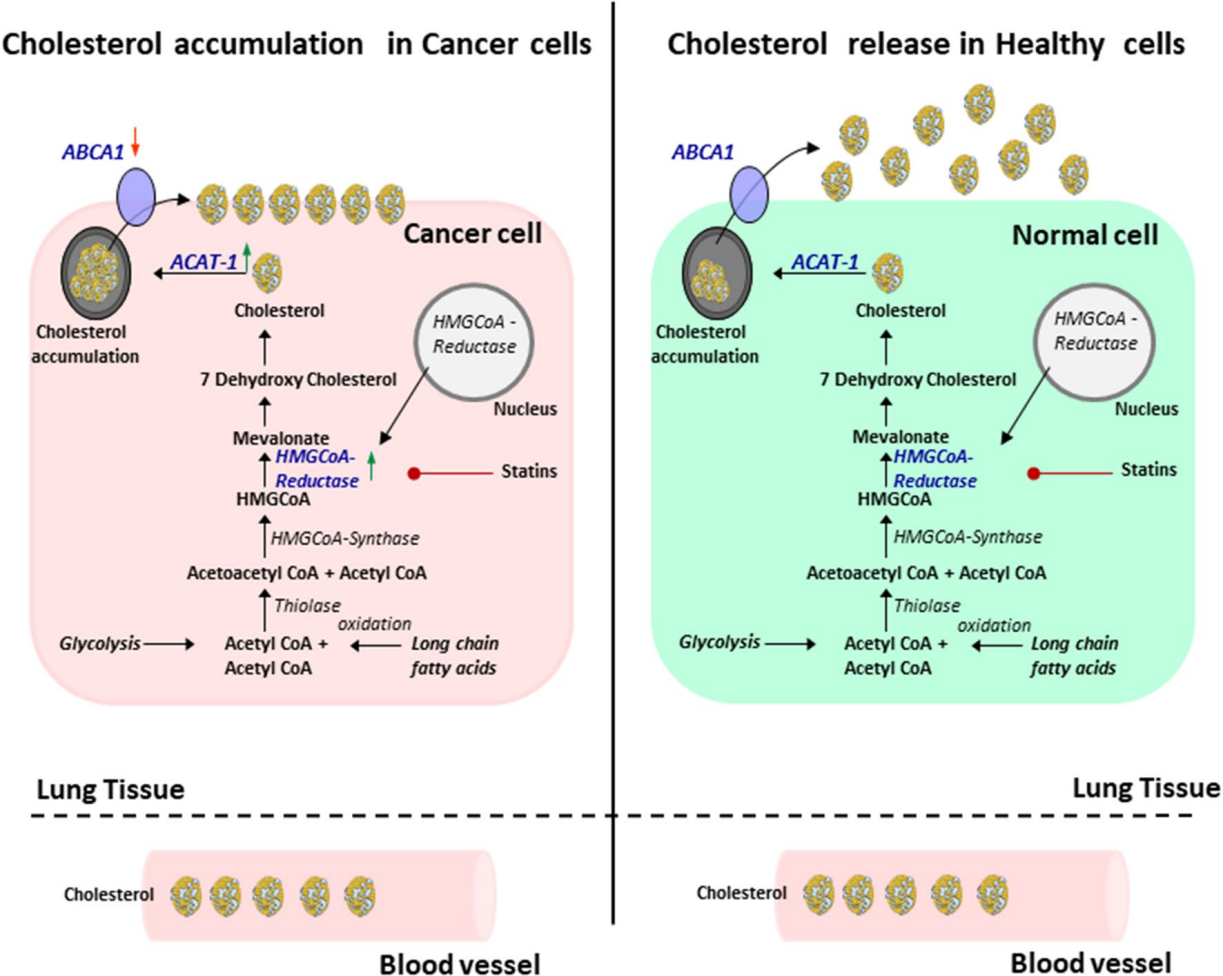
Overview of cholesterol metabolism in cancer and in normal cells. The cholesterol metabolism has many important biological functions in normal cells: Among other things, excess cellular cholesterol is converted to cholesteryl esters by the enzyme acyl-coenzyme A: Cholesterol acyltransferase (ACAT) 1 and is then removed from the intracellular spaces. In tumor cells however, the cholesterol is accumulated into the cells which then leads to increased tumor cell growth^15–17^. Parts of the figure were drawn by using pictures from Servier Medical Art. Servier Medical Art by Servier is licensed under a Creative Commons Attribution 3.0 Unported License (https://creativecommons.org/licenses/by/3.0/).

Altogether, the latter data indicate that in cancer cells there is a dysregulation of the cholesterol metabolism due to increased HMGCR and ACAT1 expression and decreased extracellular cholesterol transport resulting in cholesterol accumulation into cancer cells. To further analyze this possibility we measured the expression of the cholesterol transporter ABCA1 responsible of the egression of cholesterol from cells. Here we found a downregulation of ABCA1 mRNA/HPRT in the tumoral region of the lung as compared to the control region in patients with LUAD (Fig. 5i), especially in advanced grade 2 and 3 (Fig. 5j) and all TNM stages (Supplementary Fig. S5c). These results unravel that the lung tumor progression is strictly dependent by the cancer cells’ cholesterol metabolism and egression from tumor cells rather than the absolute level of cholesterol in the blood (Fig. 6).

## Discussion

Current immunotherapies have improved the life of patients with lung cancer. However, combined therapeutic strategies are needed to extend the success of this therapy. One problem is nutrition which can be imbalanced and thus might support the tumor growth^37,38^. We recently reported our investigation on the role of blood glucose in our cohort of patients with LUAD^4^. Other studies also demonstrated that glucose restriction in the diet resulted in decreased tumor development in a murine model of lung cancer^4,39^. Following up this observation, we asked about the role of lipids in the same cohort of patients with LUAD^3^. In this study we surprisingly did not find any difference in the level of lipids such as triglycerides and cholesterol in the serum from patients with LUAD as compared to control subjects analyzed^40^. However, our cohort of patients with lung cancer had a higher BMI as compared to control subjects. Moreover, by comparing the BMI with the triglycerides we found a direct correlation between these two values in the tumor patients but not in the control subjects. These data support a contribution of the triglycerides to the BMI in lung cancer. Obesity is suspected to be a risk for cancer. However, it is not clear if obesity per se is linked to the tumor growth. In our cohort we could not find any correlation between triglycerides and tumor diameter (Supplementary Fig. S3a,b). To continue our investigation, we analyzed HDL cholesterol and BMI and found an inverse correlation both in control and in patients with lung cancer^41^. Moreover, when we correlated the total blood cholesterol with the tumor diameter of the patients with lung cancer, we also found an indirect correlation. Altogether this would suggest a worse prognosis in patients with high triglycerides as compared to patients with high blood cholesterol.

Blood cholesterol is one of the main risks for ischemic heart disease and ischemic stroke. However, it is important to analyze the lipid fractions including HDL and non-HDL (LDL) cholesterol. These fractions have been classified as good and bad-fat, respectively, to indicate their effect on cardiovascular disease and are influenced by nutritional and life style components and medications. Consistently, we found an indirect correlation between BMI and serum levels of HDL-Cholesterol, in our patients. Moreover, we found an increased overall survival in patients with increased serum HDL cholesterol, whereas no difference in overall survival was found considering the LDL cholesterol level in serum of these patients. Furthermore, consistent with data from the literature^42^, the highest risk of cardiovascular diseases was found in patients with higher inflammation marker CRP and lower HDL cholesterol. Moreover, we found that total serum cholesterol inversely correlated with the tumor diameter as well as with serum CRP and overall survival.

We next reasoned that the blood cholesterol would not reflect the cholesterol accumulating in the lung tumor environment where tumor cell growth is supported by intracellular storage of cholesterol. To extend this investigation, we isolated mRNA from the tumoral region and tumor free region (control region) of the lung of our LUAD patients and measured HMGCR and ACAT1 mRNA, two enzymes responsible of cholesterol accumulation into the tumor cell endosomes^15^. In this investigation, we found increased mRNA levels of both HMGCR and ACAT1 in the tumoral region of the lung as compared to the control region in the lung of patients with LUAD. Finally, we found that the blood cholesterol directly correlated with ACAT1 mRNA in the control region but not in the tumoral region of the lung of patients with LUAD. Considering that the blood cholesterol level was not different between controls and LUAD patients, our data indicate that both HMGCR and ACAT1 mRNA expression in lung tumor contribute to increased intracellular cholesterol accumulation that does not correlate with cholesterol egression from the lung tumor cells. To demonstrate this point we measured the transporter molecule ABCA1 responsible for the cholesterol transport outside the cells and found it downregulated in the tumoral region. Thus, this study demonstrates that tumor progression is strictly dependent by the cancer cells’ increased cholesterol metabolism and decreased cholesterol egression from tumor cells rather than the absolute level of cholesterol in the blood.

Moreover, we have identified HMGCR and ACAT1 expression in the tumor region as possible markers for tumor progression and ABCA1 as anti-cancer gene in LUAD. These genes can easily be measured at the time of lung surgery. In conclusion, our work adds knowledge to recent literature indicating that tumor progression is associated with cancer cell softening in which cancer cells have plasma membrane enriched in cholesterol, a condition that makes tumor cells resistant to the cytotoxic effect of T cells. This indicates that, non-HDL cholesterol reduction in the diet would also increase the effect of current immunotherapy^18^. Further, detailed analysis on fraction of cholesterol accumulation in cancer cells of these patients should be performed in the future to improve current immunotherapy for lung cancer.

## Supplementary Information

Supplementary Information 1.

Supplementary Figure 1.

Supplementary Figure 2.

Supplementary Figure 3.

Supplementary Figure 4.

Supplementary Figure 5.

## Data Availability

Moreover, the raw data analysed during this study are included in the supplementary file in the tables.
