# Supplementary material for: Contribution of serum lipids and cholesterol cellular metabolism in lung cancer development and progression
Source: Sci Rep. 2023 Apr 6;13:5662. doi: 10.1038/s41598-023-31575-y (PMC10079859; doi:10.1038/s41598-023-31575-y)
Supplement: Supplementary file 1 — Supplementary Information 1. [file 41598_2023_31575_MOESM1_ESM.docx]

**Contribution of serum lipids and cholesterol cellular metabolism in lung cancer development and progression**

Philipp Hartmann^1,*^, Denis I. Trufa ^2^, Katja Hohenberger^1^, Patrick Tausche^1^, Sonja Trump^1^, Susanne Mittler^1^, Carol I. Geppert ^3^, J. Ralf J.Rieker ^3^, Oliver Schieweck^4^, Horia Sirbu^2,&^, Arndt Hartmann^3,&^, and Susetta Finotto ^1,&,**^

^1^ Department of Molecular Pneumology, Friedrich-Alexander-Universität (FAU) Erlangen- Nürnberg, Universitätsklinikum Erlangen, 91052 Erlangen, Germany
^2^ Department of Thoracic Surgery, Friedrich-Alexander-Universität (FAU) Erlangen-Nürnberg, Universitätsklinikum Erlangen, 91052 Erlangen, Germany
^3^ Institute of Pathology, Friedrich-Alexander-Universität (FAU) Erlangen-Nürnberg, Universitätsklinikum Erlangen, 91052 Erlangen, Germany
^4^ Laboratory of Clinic Medicine, Friedrich-Alexander-Universität (FAU) Erlangen-Nürnberg, Universitätsklinikum Erlangen, 91052 Erlangen, Germany

^&^ Comprehensive Cancer Center Erlangen-EMN (CCC ER-EMN), Erlangen, Germany

*The present work was performed in fulfilment of the requirements for obtaining the degree “Dr. med. dent.” for Philipp Hartmann


**Corresponding author
Prof. Susetta Finotto, PhD
Laboratories of Cellular and Molecular Lung Immunology
Department of Molecular Pneumology
Friedrich-Alexander-Universität Erlangen-Nürnberg
Hartmannstraße 14
91052 Erlangen, Germany
Phone: 0049-9131-8535883
Email: [Susetta.Finotto@uk-erlangen.de](mailto:Susetta.Finotto@uk-erlangen.de)

**Declaration of Interest**The authors declare they do not have conflict of interest on the matter described in this manuscript.

**Introduction
Role of lipids in disease**Approximately 7.2 million deaths are caused annually by cardiovascular disease (CVD) and thus by atherosclerosis, with increasing prevalence [1]. Risk factors include, among others, disorders of lipid metabolism, which are characterized by high serum levels of triglycerides or cholesterol or both [2]. With regard to the lipemic profile, the measurement of total triglycerides, cholesterol, LDL- (low-density lipoprotein) cholesterol and HDL- (high-density-lipoprotein) cholesterol is recommended for initial medical examination [3].

Triglycerides, consisting of the triple alcohol glycerol and three unsaturated or saturated fatty acids, have an average atomic mass of around 875 Da and are mainly found in adipose tissue. Transport in blood occurs bound to apolipoproteins in the form of triglyceride-rich lipoproteins (TRL) such as chylomicrons (postprandial) or other lipoprotein particles. After hydrolysis of dietary triacylglycerides and cholesteryl esters, free fatty acids and cholesterol are absorbed through enteric microvilli, where chylomicrons are formed and released into the venous blood system, passing through the ductus thoracicus and bypassing the liver. Subsequently, endothelium-associated lipoprotein lipase releases free fatty acids to supply muscle cells and adipose tissue, after which chylomicron residues are taken up by the liver [4-6]. In order to supply body tissues with cholesterol (cholesterol is an essential structural component of steroid hormones and cell membranes) and fatty acids, the liver releases VLDL (very low-density lipoprotein particle) and IDL (intermediate-density lipoprotein particle), which are composed of *e. g.* phospholipids, cholesteryl esters, triacylglycerides and the protein fraction apoB (apolipoprotein B). While the formation of LDL (low-density lipoprotein particles), which is considered to be a major factor in the development of atherosclerosis, results from the processing of VLDL by lipoprotein lipase, nascent HDL (high-density lipoprotein particles) contributes to the removal of cholesterol and is generated by lipoprotein lipase-mediated lipolysis of TRLs [4-7].

In addition to food intake, endogenous synthesis is the main source of human cholesterol [7].

Lipoprotein A (Lp (a)) is considered an independent risk factor for atherosclerotic processes and stroke [8-10].

**References**

1. Benjamin, E.J., et al., *Heart Disease and Stroke Statistics-2018 Update: A Report From the American Heart Association.* Circulation, 2018. **137**(12): p. e67-e492.

2. Kemal, A., et al., *Dyslipidemia and Associated Factors Among Adult Patients on Antiretroviral Therapy in Armed Force Comprehensive and Specialized Hospital, Addis Ababa, Ethiopia.* HIV AIDS (Auckl), 2020. **12**: p. 221-231.

3. Expert Panel on Detection, E. and A. Treatment of High Blood Cholesterol in, *Executive Summary of The Third Report of The National Cholesterol Education Program (NCEP) Expert Panel on Detection, Evaluation, And Treatment of High Blood Cholesterol In Adults (Adult Treatment Panel III).* JAMA, 2001. **285**(19): p. 2486-97.

4. Wong, E., *Clinical Laboratory Diagnostics: Use and Assessment of Clinical Laboratory Results. Lothar Thomas. Frankfurt/Main, Germany: TH-Books Verlagsgeselschaft, 1998, 1727 pp., $149.00. ISBN 3-9805215-4-0.* Clinical Chemistry, 1999. **45**(4): p. 586-587.

5. Caponio, G.R., et al., *Regulation of Cholesterol Metabolism by Bioactive Components of Soy Proteins: Novel Translational Evidence.* Int J Mol Sci, 2020. **22**(1).

6. Duan, Y., et al., *Regulation of cholesterol homeostasis in health and diseases: from mechanisms to targeted therapeutics.* Signal Transduct Target Ther, 2022. **7**(1): p. 265.

7. Lu, Y., et al., *The Functional Role of Lipoproteins in Atherosclerosis: Novel Directions for Diagnosis and Targeting Therapy.* Aging Dis, 2022. **13**(2): p. 491-520.

8. Kronenberg, F. and G. Utermann, *Lipoprotein(a): resurrected by genetics.* J Intern Med, 2013. **273**(1): p. 6-30.

9. Kamstrup, P.R., et al., *Genetically elevated lipoprotein(a) and increased risk of myocardial infarction.* Jama, 2009. **301**(22): p. 2331-9.

10. Trégouët, D.A., et al., *Genome-wide haplotype association study identifies the SLC22A3-LPAL2-LPA gene cluster as a risk locus for coronary artery disease.* Nat Genet, 2009. **41**(3): p. 283-5.

**Supplementary Table S1: General Characteristics of the cohort of patients with NSCLC analyzed in this study.**

| **Patient Code** | **Age** | **Gender** | **Body weight [kg]** | **Body height [m]** | **BMI = Weight/Height²** | **Waist circum-ference [cm]** | **Histological Classification** | **Grading** | **TNM-Stadium** | **Tumor Diameter Patho (cm)** |
| --- | --- | --- | --- | --- | --- | --- | --- | --- | --- | --- |
| 44-MP | 53 | Male | 120 | 1.87 | 34.32 | 112 | ADC | G1 | IA2 | 1.5 |
| 45-MP | 78 | Male | 100 | 1.68 | 35.43 | 107 | ADC | G1 | IA3 | 2.3 |
| 107-MP | 54 | Female | 55 | 1.6 | 21.48 | - | ADC | G1 | IA2 | 1.7 |
| 118-MP | 67 | Female | 83 | 1.58 | 33.25 | - | ADC | G1 | IA2 | 1.5 |
| 146-MP | 67 | Female | 68 | 1.57 | 27.59 | - | ADC | G1 | IA | 1.8 |
| 151-MP | 61 | Male | 85 | 1.79 | 26.53 | - | ADC | G1 | IIA | 4.2 |
| 9-MP-IL35 | 84 | Female | 75 | 1.58 | 30.04 | 116 | ADC | G2 | IIIA | 2.7 |
| 17-MP | 74 | Male | 90 | 1.72 | 30.42 | - | ADC | G2 | IA3 | 2.6 |
| 23-MP | 73 | Male | 78 | 1.82 | 23.55 | 98 | ADC | G2 | IIA | 4.5 |
| 34-MP | 51 | Female | 57 | 1.68 | 20.20 | - | ADC | G2 | IA2 | 1.8 |
| 53-MP | 62 | Male | 113 | 1.91 | 30.98 | - | ADC | G2 | IA2 | 1.4 |
| 56-MP | 68 | Female | 110 | 1.65 | 40.40 | - | ADC | G2 | IB | 4 |
| 57-MP | 36 | Female | 144 | 1.71 | 49.25 | - | ADC | G2 | IB | 3.8 |
| 59-MP | 71 | Male | 65 | 1.74 | 21.47 | 92 | ADC | G2 | IIIA | 0.9 2.1 |
| 62-MP | 80 | Female | 125 | 1.65 | 45.91 | 148 | ADC | G2 | IB | 3.5 |
| 64-MP | 55 | Male | 79 | 1.78 | 24.93 | - | ADC | G2 | IVA | 3.5 |
| 69-MP | 76 | Female | 63 | 1.58 | 25.24 | - | ADC | G2 | IB | 3.1 |
| 70-MP | 70 | Female | 58 | 1.62 | 22.10 | 95 | ADC | G2 | IA3 | 2.7 |
| 73-MP | 67 | Female | 80 | 1.71 | 27.36 | - | ADC | G2 | IIA | 4.8 |
| 74-MP | 58 | Female | 71 | 1.52 | 30.73 | - | ADC | G2 | IB | 3.2 |
| 77-MP | 64 | Female | 68 | 1.68 | 24.09 | - | ADC | G2 | IA1 | 0.9 |
| 86-MP | 54 | Male | 55 | 1.76 | 17.76 | - | ADC | G2 | IVA | 1.8 |
| 88-MP | 73 | Male | 74 | 1.7 | 25.61 | - | ADC | G2 | IA3 | 2.1 |
| 91-MP | 67 | Male | 72 | 1.73 | 24.06 | 90 | ADC | G2 | IIIA | 3.6 |
| 94-MP | 77 | Female | 73 | 1.76 | 23.57 | - | ADC | G2 | IA3 | 2.2 |
| 97-MP | 73 | Male | 103 | 1.88 | 29.14 | 109 | ADC | G2 | IIB | 5 |
| 102-MP | 66 | Male | 70 | 1.83 | 20.90 | 93 | ADC | G2 | IIB | 2.1 |
| 106-MP | 79 | Male | 69 | 1.68 | 24.45 | 89 | ADC | G2 | IA2 | 1.2 |
| 108-MP | 60 | Female | 50 | 1.58 | 20.03 | - | ADC | G2 | IB | 3.6 |
| 112-MP | 74 | Female | 78 | 1.63 | 29.36 | 110 | ADC | G2 | IIIA | 1.5  1.6 |
| 124-MP | 71 | Male | 59 | 1.75 | 19.27 | 89 | ADC | G2 | IA2 | 1.2 |
| 125-MP | 56 | Female | 71 | 1.62 | 27.05 | 99 | ADC | G2 | IIB | 7.9 |
| 126-MP | 62 | Male | 95 | 1.75 | 31.02 | 106 | ADC | G2 | IIIA | 2.3 |
| 140-MP | 70 | Female | 83 | 1.57 | 33.67 | - | ADC | G2 | IA3 | 2.1 |
| 141-MP | 48 | Female | 67 | 1.72 | 22.65 | - | ADC | G2 | IB | 3.8 |
| 143-MP | 82 | Male | 86 | 1.73 | 28.73 | - | ADC | G2 | IIIA | 2.7  1.5 |
| 149-MP | 69 | Male | 75 | 1.82 | 22.64 | - | ADC | G2 | IA2 | 1.2 |
| 150-MP | 64 | Male | 98 | 1.83 | 29.26 | - | ADC | G2 | IA3 | 2.3 |
| 155-MP | 75 | Male | 80 | 1.71 | 27.36 | - | ADC | G2 | IA2 | 1.3 |
| 3-MP-IL35 | 79 | Male | 85 | 1.76 | 27.44 | 105 | ADC | G3 | IIA | 5 |
| 15-MP-IL35 | 63 | Male | 95 | 1.74 | 31.38 | 105 | ADC | G3 | IA3 | 2.5 |
| 16-MP | 70 | Female | 80 | 1.7 | 27.68 | 112 | ADC | G3 | IIB | 1.8 2.8 |
| 20-MP | 60 | Male | 72 | 1.85 | 21.04 | 96 | ADC | G3 | IA3 | 2.7 |
| 22-MP | 68 | Male | 89.5 | 1.81 | 27.32 | - | ADC | G3 | IIIA | 7 |
| 26-MP | 52 | Female | 73 | 1.6 | 28.52 | - | ADC | G3 | IVA | 1.3 |
| 27-MP | 70 | Female | 79 | 1.73 | 26.40 | - | ADC | G3 | IA2 | 1.4 |
| 28-MP | 76 | Male | 85 | 1.73 | 28.40 | - | ADC | G3 | IA2 | 1.2 |
| 32-MP | 60 | Female | 66 | 1.58 | 26.44 | - | ADC | G3 | IIIA | 4.4 |
| 35-MP | 72 | Female | 80 | 1.5 | 35.56 | - | ADC | G3 | IA3 | 3 |
| 39-MP | 65 | Male | 95 | 1.68 | 33.66 | 106 | ADC | G3 | IIB | 6 |
| 40-MP | 82 | Male | 51 | 1.7 | 17.65 | 88 | ADC | G3 | IIB | 1.8 |
| 43-MP | 72 | Female | 85 | 1.66 | 30.85 | 102 | ADC | G3 | IIIA | 4 |
| 51-MP | 61 | Male | 80 | 1.8 | 24.69 | - | ADC | G3 | IVA | 2.3 2.5 |
| 55-MP | 64 | Female | 75 | 1.6 | 29.30 | - | ADC | G3 | IIIA | 1.8 |
| 68-MP | 42 | Male | 82 | 1.76 | 26.47 | - | ADC | G3 | IIIA | 8.5 |
| 71-MP | 67 | Male | 90 | 1.72 | 30.42 | - | ADC | G3 | IB | 3.7 |
| 78-MP | 80 | Female | 69 | 1.62 | 26.29 | - | ADC | G3 | IIIA | 2.1 |
| 80-MP | 62 | Male | 108 | 1.76 | 34.87 | 112 | ADC | G3 | IIB | 5.4 |
| 81-MP | 61 | Male | 79 | 1.74 | 26.09 | - | ADC | G3 | IA2 | 1.6 |
| 83-MP | 60 | Female | 57 | 1.56 | 23.42 | 91 | ADC | G3 | IVA | 5.5 |
| 89-MP | 61 | Female | 59 | 1.54 | 24.88 | - | ADC | G3 | IIIA | 3.7 |
| 93-MP | 66 | Male | 87 | 1.73 | 29.07 | - | ADC | G3 | IIB | 2.8 |
| 98-MP | 55 | Female | 43 | 1.58 | 17.22 | 76 | ADC | G3 | IIIA | 2.9 |
| 99-MP | 62 | Male | 56 | 1.72 | 18.93 | - | ADC | G3 | IIIA | 3.5  1.5 |
| 103-MP | 70 | Female | 62 | 1.59 | 24.52 | - | ADC | G3 | IB | 3.9 |
| 105-MP | 77 | Male | 73 | 1.68 | 25.86 | - | ADC | G3 | IA2 | 1.8 |
| 110-MP | 77 | Female | 64 | 1.58 | 25.64 | 100 | ADC | G3 | IIIB | 7.2 |
| 113-MP | 68 | Male | 88 | 1.78 | 27.77 | - | ADC | G3 | IIB | 7.5 |
| 121-MP | 59 | Male | 81 | 1.8 | 25.00 | - | ADC | G3 | IIIA | 12.5 |
| 122-MP | 61 | Female | 116 | 1.82 | 35.02 | - | ADC | G3 | IA3 | 2.8 |
| 129-MP | 65 | Female | 70 | 1.69 | 24.51 | - | ADC | G3 | IIIB | 3.3 |
| 132-MP | 64 | Male | 82 | 1.84 | 24.22 | - | ADC | G3 | IB | 3.5 |
| 133-MP | 67 | Female | 86 | 1.6 | 33.59 | - | ADC | G3 | IA2 | 1.9 |
| 134-MP | 53 | Female | 73 | 1.78 | 23.04 | - | ADC | G3 | IIB | 3.5 |
| 135-MP | 54 | Female | 66 | 1.65 | 24.24 | - | ADC | G3 | IIIA | 9.5 |
| 137-MP | 60 | Female | 73 | 1.7 | 25.26 | - | ADC | G3 | IIIA | 7.3 |
| 139-MP | 70 | Female | 79 | 1.65 | 29.02 | - | ADC | G3 | IA2 | 1.2 |
| 147-MP | 59 | Female | 46 | 1.6 | 17.97 | - | ADC | G3 | IB | 0.8 |
| 19-MP | 55 | Female | 66 | 1.67 | 23.67 | 96 | ADC | - | IIB | 7 |
| 72-MP | 53 | Male | 76.5 | 1.75 | 24.98 | - | ADC | - | IIIC | 3.5 |
| 136-MP | 66 | Male | 104 | 1.9 | 28.81 | - | ADC | - | IIB | 2.2 |

**Supplementary Table S2: Lipid values of the cohort of patients with NSCLC analyzed in this study.**

| **Patient Code** | **Total cholesterol before [mg/dl]** | **HDL cholesterol before [mg/dl]** | **LDL cholesterol before[mg/dl]** | **Triglycerides before [mg/dl]** | **Lipoprotein a before [mg/dl]** |
| --- | --- | --- | --- | --- | --- |
| 44-MP | 107 | 32 | 58 | 115 | 7.9 |
| 45-MP | 187 | 30 | 128 | 161 | 13.4 |
| 107-MP | 296 | 66 | 186 | 173 | 7.9 |
| 118-MP | 253 | 55 | 157 | 182 | 15.7 |
| 146-MP | 183 | 63 | 108 | 77 | 7.9 |
| 151-MP | 167 | 47 | 103 | 169 | 94.7 |
| 9-MP-IL35 | - | - | - | - | - |
| 17-MP | 103 | 22 | 72 | 159 | 7.9 |
| 23-MP | - | - | - | - | - |
| 34-MP | - | - | - | - | - |
| 53-MP | 156 | 32 | 86 | 139 | 7.9 |
| 56-MP | 134 | 38 | 86 | 95 | 71.6 |
| 57-MP | 145 | 32 | 90 | 131 | 7.9 |
| 59-MP | 59 | 9 | 41 | 128 | 31.6 |
| 62-MP | 138 | 37 | 81 | 147 | 7.9 |
| 64-MP | 180 | 28 | 127 | 147 | 7.9 |
| 69-MP | - | - | - | - | - |
| 70-MP | 118 | 34 | 58 | 205 | 7.9 |
| 73-MP | 165 | 52 | 94 | 110 | 7.9 |
| 74-MP | 160 | 29 | 111 | 125 | 7.9 |
| 77-MP | 208 | 61 | 127 | 57 | 7.9 |
| 86-MP | 158 | 59 | 79 | 94 | 10.4 |
| 88-MP | 170 | 41 | 105 | 97 | 7.9 |
| 91-MP | 180 | 83 | 80 | 72 | 30.2 |
| 94-MP | 211 | 49 | 134 | 111 | 7.9 |
| 97-MP | 150 | 29 | 98 | 188 | 7.9 |
| 102-MP | 152 | 38 | 96 | 84 | 13.9 |
| 106-MP | 151 | 41 | 90 | 112 | 7.9 |
| 108-MP | 217 | 48 | 141 | 98 | 9.7 |
| 112-MP | 178 | 41 | 108 | 127 | 7.9 |
| 124-MP | 185 | 40 | 123 | 84 | 14.5 |
| 125-MP | 129 | 26 | 87 | 110 | 7.9 |
| 126-MP | 180 | 37 | 124 | 139 | 76.2 |
| 140-MP | 190 | 46 | 121 | 126 | 7.9 |
| 141-MP | 211 | 49 | 138 | 83 | 7.9 |
| 143-MP | 181 | 39 | 122 | 142 | 14.4 |
| 149-MP | 165 | 80 | 72 | 94 | 18.7 |
| 150-MP | 257 | 34 | 186 | 150 | 7.9 |
| 155-MP | 137 | 37 | 87 | 168 | 7.9 |
| 3-MP-IL35 | - | - | - | - | - |
| 15-MP-IL35 | - | - | - | - | - |
| 16-MP | - | - | - | - | - |
| 20-MP | - | - | - | - | - |
| 22-MP | - | - | - | - | - |
| 26-MP | - | - | - | - | - |
| 27-MP | 161 | 28 | 117 | 154 | 28.9 |
| 28-MP | - | - | - | - | - |
| 32-MP | - | - | - | - | - |
| 35-MP | - | - | - | - | - |
| 39-MP | 112 | 31 | 65 | 110 | 7.9 |
| 40-MP | 204 | 43 | 140 | 100 | 7.9 |
| 43-MP | 202 | 72 | 113 | 83 | 38.7 |
| 51-MP | 151 | 23 | 105 | 161 | 7.9 |
| 55-MP | 222 | 64 | 135 | 105 | 14.2 |
| 68-MP | 115 | 27 | 72 | 87 | 7.9 |
| 71-MP | 121 | 36 | 71 | 152 | 14.3 |
| 78-MP | 223 | 61 | 135 | 90 | 13.7 |
| 80-MP | 206 | 27 | 141 | 204 | 7.9 |
| 81-MP | 151 | 34 | 97 | 100 | 7.9 |
| 83-MP | 152 | 38 | 92 | 115 | 7.9 |
| 89-MP | 178 | 55 | 103 | 77 | 11.1 |
| 93-MP | 237 | 42 | 161 | 171 | 7.9 |
| 98-MP | 206 | 99 | 86 | 87 | 11.7 |
| 99-MP | 174 | 34 | 117 | 115 | 19.3 |
| 103-MP | 208 | 61 | 118 | 139 | 66 |
| 105-MP | 180 | 61 | 90 | 180 | 7.9 |
| 110-MP | 170 | 57 | 94 | 106 | 13.1 |
| 113-MP | 142 | 46 | 68 | 147 | 7.9 |
| 121-MP | 216 | 40 | 148 | 118 | 14.6 |
| 122-MP | 222 | 34 | 125 | 238 | 7.9 |
| 129-MP | 109 | 31 | 66 | 88 | 7.9 |
| 132-MP | 175 | 42 | 116 | 93 | 7.9 |
| 133-MP | 250 | 45 | 172 | 182 | 7.9 |
| 134-MP | 213 | 66 | 126 | 95 | 66.5 |
| 135-MP | 116 | 51 | 57 | 60 | 7.9 |
| 137-MP | 170 | 45 | 109 | 86 | 102.9 |
| 139-MP | 286 | 61 | 200 | 178 | 169.3 |
| 147-MP | 210 | 87 | 105 | 129 | 7.9 |
| 19-MP | - | - | - | - | - |
| 72-MP | 107 | 26 | 60 | 141 | 7.9 |
| 136-MP | 206 | 68 | 119 | 144 | 13 |

**Supplementary Table S3 (cohort of patients with NSCLC analyzed in this study).**

| **Patient Code** | **Smoking (PJ)** | **Albumin before [g/l]** | **CRP before [mg/l] <5** | **CRP after [mg/dl]** | **Ly absolut before x 10^3/ul** | **Ly before %** | **FEV1 %** | **FVC %** | **Glucose [mg/dl]** |
| --- | --- | --- | --- | --- | --- | --- | --- | --- | --- |
| 44-MP | 70 | 45.5 | 0.8 | 66.9 | 2.13 | 30.6 | 87.8 | 85.9 | 108 |
| 45-MP | 0 | - | 0.6 | 163.2 | 1.92 | 31.8 | 84.2 | 106.3 | 94 |
| 107-MP | 20 | 45.8 | 2.6 | 229.7 | 2.91 | 25.5 | - | - | 97 |
| 118-MP | 50 | 44.3 | 5.4 | 31.5 | 2.07 | 22.5 | 87.1 | 100.4 | - |
| 146-MP | 40 | 39.9 | 1.6 | 28.8 | 1.43 | 25.4 | 80 | 94 | 101 |
| 151-MP | 15 | - | 0.3 | 58.4 | 1.46 | 30.6 | 99 | 105 | 98 |
| 9-MP-IL35 | 0 | 38.8 | 2 | 62.4 | 2.14 | 29.9 | 85.5 | 98.2 | 83 |
| 17-MP | 70 | - | 1.7 | 135.2 | 1.87 | 28.1 | 93 | 84 | 110 |
| 23-MP | 75 | 41.9 | 2.6 | 159.2 | 1.49 | 21.3 | 116.8 | 118.2 | 101 |
| 34-MP | 45 | 45.2 | - | 58.2 | 1.59 | 30.2 | 40.1 | 90 | 84 |
| 53-MP | 30 | 43.8 | 5.1 | 93.9 | 2.32 | 29.4 | 52.8 | 65.7 | 101 |
| 56-MP | 0 | 45.4 | 4.6 | 200.9 | 1.68 | 23.4 | 99.2 | 95.1 | 112 |
| 57-MP | - | 40.3 | 8.2 | 44.3 | 2.67 | 30.7 | 108.3 | 114.6 | 100 |
| 59-MP | 40 | 22.9 | 32.2 | 113.2 | 0.48 | 6.5 | 88 | - | 161 |
| 62-MP | 0 | 28.5 | 8.8 | 142.3 | 1.78 | 31 | 100.2 | - | 89 |
| 64-MP | 30 | 41.5 | 8.5 | 231.9 | 1.85 | 24.2 | 68 | 95.3 | 94 |
| 69-MP | - | 44.6 | 2.3 | 114.7 | 1.36 | 23.1 | 97.4 | 101 | 81 |
| 70-MP | 100 | - | - | 171.4 | 2.44 | 30.5 | 59.7 | - | - |
| 73-MP | 22 | 46.3 | 1 | 216.2 | 2.29 | 32.4 | 99.9 | - | 93 |
| 74-MP | - | - | 1.4 | 92.7 | 1.63 | 27.4 | 124 | 120 | - |
| 77-MP | 45 | 45.8 | 2.4 | 47.3 | 2 | 31.2 | 96.6 | 102 | 259 |
| 86-MP | 40 | 44.5 | 0.2 | 209.4 | 1.12 | 6.3 | 85.5 | 110.7 | 65 |
| 88-MP | 10 | 44.4 | 0.5 | 78.4 | 1.1 | 21 | 98.8 | 76.5 | 102 |
| 91-MP | 0 | 46.3 | 0.8 | 180.8 | 1.15 | 17.2 | - | - | 101 |
| 94-MP | 35 | 41.1 | 0.8 | 55.3 | 1.64 | 28 | 72.7 | 79.4 | 108 |
| 97-MP | 0 | 43.5 | <0.2 | 75.2 | 1.91 | 32.9 | 94.1 | 103.2 | 127 |
| 102-MP | 18 | 38.2 | 6.2 | 190.7 | 2.7 | 36.7 | - | - | 101 |
| 106-MP | 60 | - | - | 111.1 | - | - | 65.5 | 86.8 | - |
| 108-MP | 40 | 46.9 | 9 | 44.3 | 2.62 | 21.3 | 74 | - | 116 |
| 112-MP | 0 | 42.3 | 2.8 | 113.1 | 2.52 | 32.3 | 88.9 | 87.1 | 101 |
| 124-MP | 75 | 43.3 | 0.8 | 109.3 | 1.83 | 25.1 | 84.2 | 102.8 | - |
| 125-MP | 9 | 38.7 | 49.8 | 134.4 | 3.54 | 19.1 | 94.5 | 92.5 | 112 |
| 126-MP | 40 | 42.4 | 2.7 | 169.1 | 1.37 | 21.8 | 95.7 | 121.5 | - |
| 140-MP | 5 | - | 6.2 | 179.1 | 3.14 | 24.1 | 96 | 91 | 112 |
| 141-MP | 30 | 39.4 | 0.7 | 75.3 | 1.4 | 23.6 | 80 | 118 | 100 |
| 143-MP | 0 | - | 11.8 | 86.3 | 1.23 | 15.7 | 121 | 102 | - |
| 149-MP | 84 | 38.1 | 0.9 | 30.9 | 1.24 | 15.1 | 40 | 57 | 109 |
| 150-MP | 100 | 42.4 | 3.6 | 64.8 | 2.56 | 26 | 58 | 84 | 91 |
| 155-MP | 1,6 | - | 1.1 | 120.9 | 0.47 | 8.2 | 82 | 75 | - |
| 3-MP-IL35 | 60 | - | 24.6 | 138 | 1.54 | 68.2 | 46.1 | 54.1 | - |
| 15-MP-IL35 | 100 | - | 24.4 | 183.8 | 1.93 | 15.1 | 96.5 | 76.8 | 125 |
| 16-MP | 15 | - | 8.1 | 104.3 | 1.44 | 8.6 | 66.4 | 77.3 | 93 |
| 20-MP | 65 | 42.1 | 2.2 | 52.2 | 2.11 | 20.1 | - | - | 110 |
| 22-MP | 82 | 45.3 | 4.7 | 112.2 | 1.52 | 27.9 | 83 | 78.1 | 117 |
| 26-MP | 50 | 39.8 | 1.5 | 73 | 1.08 | 13 | 105.8 | 114.1 | 104 |
| 27-MP | 50 | - | 1.4 | 73.7 | 2.73 | 42.5 | - | - | 93 |
| 28-MP | 60 | 41.7 | 3.5 | 215 | 1.22 | 20.4 | 45 | 52 | 100 |
| 32-MP | 30 | 46.1 | 2 | 40 | 1.25 | 25.1 | 99.8 | 105.4 | - |
| 35-MP | 0 | 42.5 | 1.4 | 52.1 | 1.46 | 29.9 | 135.2 | 125.5 | 102 |
| 39-MP | 42 | 38.3 | 18.2 | 178.7 | 1.36 | 10.1 | 77.4 | 81.2 | 134 |
| 40-MP | 100 | 43.4 | 4.9 | 138.4 | 1.32 | 14.9 | 74.9 | 90.1 | 86 |
| 43-MP | secondhand smoker  25 years | 41.5 | 1.5 | 25.2 | 1.21 | 20.8 | 94.4 | 97.3 | 96 |
| 51-MP | - | 38 | - | 3.4 | 2.32 | 26.2 | 76.3 | 87.2 | 96 |
| 55-MP | 40 | 42.8 | 3 | 78.6 | 3.51 | 39.8 | 82 | 98.6 | 92 |
| 68-MP | 22 | 37 | 130 | 43.1 | 1.77 | 24.7 | 103.7 | 96.3 | 91 |
| 71-MP | 75 | 42.8 | 16.6 | 159.9 | 1.48 | 15.8 | 90.3 | 95.1 | 109 |
| 78-MP | 0 | 42.6 | 0.5 | 34.8 | 0.82 | 14.6 | 85.2 | 89 | 102 |
| 80-MP | 28 | 41.3 | 9 | 239,2 | 1.63 | 18.5 | 62.8 | 63.8 | 106 |
| 81-MP | 46 | 41.2 | 1.1 | 73.2 | 3.27 | 48.7 | 102.9 | 115.8 | 98 |
| 83-MP | 0 | 45.3 | 9.7 | 201.6 | 1.14 | 14.7 | 113.6 | 105.5 | 98 |
| 89-MP | 0 | 45.7 | 3.6 | 67.4 | 1.26 | 14 | 85.8 | 101.6 | 94 |
| 93-MP | 32,5 | 40.7 | 2.6 | 111.2 | 2.04 | 35.2 | 115.7 | 120 | 104 |
| 98-MP | 30 | 42.9 | 0.6 | 44.2 | 2.1 | 27 | 74.8 | 97.1 | 102 |
| 99-MP | - | 39.3 | 38.8 | 394.8 | 1.07 | 6 | 68.9 | 103.7 | - |
| 103-MP | 100 | 41.5 | 1.1 | 105.3 | 2.01 | 28.2 | 81.2 | 110.6 | 108 |
| 105-MP | 35 | - | - | 74.5 | 0.49 | 9.4 | 64.6 | - | - |
| 110-MP | 45 | - | 56.1 | 125.4 | 1.26 | 9.1 | 95.7 | 86.4 | - |
| 113-MP | 52,5 | 35.4 | 5.7 | 88.4 | 0.77 | 9.5 | 80.5 | 74.2 | 166 |
| 121-MP | 67,5 | 38.3 | 44.6 | 92.1 | 1.14 | 23.3 | - | - | - |
| 122-MP | 50 | 38 | 22.9 | 128 | 1.7 | 22.1 | 54.3 | 67.2 | 94 |
| 129-MP | 45 | - | 1.6 | 119.5 | 2.63 | 41.9 | 62 | 72 | - |
| 132-MP | 100 | - | 24.3 | 177.9 | 0.99 | 17.3 | 90 | 92 | - |
| 133-MP | 20 | - | 4.7 | 77.2 | 0.88 | 18.7 | 94 | 102 | - |
| 134-MP | 18 | - | - | 98.3 | 1.23 | 6 | - | - | - |
| 135-MP | 23 | 42.1 | 1.7 | 31.7 | 1.76 | 24.8 | 51 | 96 | 99 |
| 137-MP | 40 | 34.3 | 52.9 | 121.5 | 1.54 | 24.2 | 56 | 91 | 61 |
| 139-MP | 48 | 37.7 | 6.7 | 111.6 | 1.71 | 28.5 | 59 | 78 | 96 |
| 147-MP | 20 | - | 27.9 | 86 | 1.51 | 20.9 | 65 | 59 | - |
| 19-MP | 30 | - | 14 | 227 | 1.04 | 15.9 | 65.1 | 69.4 | - |
| 72-MP | - | 40.6 | 8.9 | 145.7 | 2.66 | 33.2 | 43.5 | 50.2 | 104 |
| 136-MP | 20 | 38.6 | 1.1 | 31 | 2.24 | 28.3 | 95 | 96 | 100 |

**Supplementary Table S4: General Characteristics of the cohort of control patients analyzed in this study.**

| **Patient Code** | **Age** | **Gender** | **Body Weight [kg]** | **Body Height [m]** | **BMI = Weight/ Height²** | **Smoking [PJ]** |
| --- | --- | --- | --- | --- | --- | --- |
| CN 1 | 55 | female | 68 | 1.66 | 24.68 | 20 |
| CN 2 | 61 | Male | 56 | 1.7 | 19.38 | 50 |
| CN 3 | 50 | Male | 69 | 1.7 | 23.88 | 35 |
| CN 6 | 53 | Male | 83 | 1.83 | 24.78 | 2 |
| CN 7 | 37 | female | 48 | 1.76 | 15.50 | 8 |
| CN 8 | - | - | - | - | - | - |
| CN 9 | 51 | female | 66 | 1.6 | 25.78 | 35 |
| CN 10 | 53 | Male | 84 | 1.85 | 24.54 | 2.5 |
| CN 11 | 24 | Male | 75.5 | 1.95 | 19.86 | 0 |
| CN 12 | 47 | Male | 73.2 | 1.87 | 20.93 | 25 |
| CN 13 | 18 | Male | - | - | - | - |
| CN 14 | 21 | Male | - | - | - | - |
| CN 15 | - | female | - | - | - | - |
| iK 6 | 24 | Male | 89 | 1.86 | 25.73 | 0 |
| iK 7 | 52 | Male | 88 | 1.79 | 27.46 | 7 |
| iK 8 | 24 | Male | 89.5 | 1.84 | 26.44 | 4 |
| iK11 | 46 | Male | 76 | 1.75 | 24.82 | 36 |
| iK12 | 24 | Male | 78 | 1.77 | 24.90 | 5 |
| iK13 | 34 | female | 61 | 1.71 | 20.86 | 0 |
| iK14 | 56 | female | 68 | 1.76 | 21.95 | 0 |
| C 501 | 33 | female | 62 | 1.64 | 23.10 | - |
| C 507 | 33 | female | 54 | 1.62 | 20.60 | - |
| C 508 | 29 | female | 45 | 1.57 | 18.30 | - |
| C 512 | 44 | female | 68 | 1.76 | 22.00 | -- |
| C 514 | 62 | Male | 80 | 1.72 | 27.00 | - |
| C 515 | 64 | Male | 97 | 1.82 | 29.30 | - |
| C 517 | 26 | Male | 103 | 1.93 | 27.70 | - |
| C 518 | 23 | female | 71 | 1.79 | 22.20 | - |
| C 520 | 63 | female | 53 | 1.69 | 18.60 | - |
| C 521 | 53 | female | 66 | 1.63 | 24.80 | - |
| C 523 | 55 | Male | 109 | 1.96 | 28.40 | - |
| C 524 | 21 | Male | 85 | 1.88 | 24.00 | - |
| C 525 | 22 | female | 59 | 1.71 | 20.20 | - |
| C 526 | 31 | Male | 91 | 1.72 | 30.80 | - |
| C 529 | 25 | Male | 76 | 1.9 | 21.10 | - |
| C 531 | 30 | Male | 90 | 1.73 | 30.10 | - |
| C 534 | 28 | female | 45 | 1.62 | 17.10 | - |
| C 536 | 61 | female | 63 | 1.68 | 22.30 | - |
| C 547 | 24 | Male | 88 | 1.84 | 26.00 | - |

**Supplementary Table S5: Lipid values of the cohort of control patients analyzed in this study.**

| **Patient Code** | **Total cholesterol before [mg/dl]** | **HDL cholesterol before [mg/dl] >40** | **LDL cholesterol before[mg/dl] <116** | **Triglycerides before [mg/dl]** | **Lipoprotein a before [mg/dl]** |
| --- | --- | --- | --- | --- | --- |
| CN 1 | 273 | 53 | 186 | 188 | 71 |
| CN 2 | 158 | 54 | 91 | 95 | 32.6 |
| CN 3 | 217 | 34 | 154 | 176 | 70.6 |
| CN 6 | 78 | 28 | 40 | 83 | 7.9 |
| CN 7 | 196 | 68 | 111 | 82 | 6.2 |
| CN 8 | 170 | 53 | 101 | 81 | 17.2 |
| CN 9 | 228 | 69 | 133 | 84 | 6.2 |
| CN 10 | 188 | 50 | 115 | 119 | 7.9 |
| CN 10 3nOP | 180 | 63 | 99 | 95 | 11.6 |
| CN 11 | 162 | 43 | 98 | 115 | 39.1 |
| CN 12 | 142 | 35 | 92 | 85 | 7.8 |
| CN 13 | 136 | 35 | 80 | 112 | 7.9 |
| CN 14 | 125 | 21 | 93 | 133 | 21.3 |
| CN 15 | 307 | 58 | 205 | 123 | 195.8 |
| iK 6 | 159 | 57 | 92 | 75 | 6.6 |
| iK 7 | 232 | 49 | 156 | 78 | 5.8 |
| iK 8 | 179 | 50 | 107 | 96 | 58.2 |
| iK11 | 173 | 64 | 97 | 41 | 60.9 |
| iK12 | 134 | 49 | 75 | 69 | 57.7 |
| iK13 | 204 | 45 | 138 | 121 | 46.6 |
| iK14 | 256 | 101 | 136 | 67 | 7.9 |
| C 501 | 254 | 46 | 174 | 78 | 7.9 |
| C 507 | 213 | 86 | 110 | 73 | 7.9 |
| C 508 | 168 | 63 | 97 | 68 | 15.2 |
| C 512 | 118 | 59 | 79 | 49 | 6.4 |
| C 514 | 189 | 49 | 116 | 81 | 7.9 |
| C 515 | 225 | 46 | 147 | 99 | 50.8 |
| C 517 | 148 | 40 | 92 | 106 | 47.3 |
| C 518 | 160 | 78 | 77 | 65 | 7.4 |
| C 520 | 182 | 82 | 90 | 46 | 35.5 |
| C 521 | 235 | 80 | 135 | 50 | 21.58 |
| C 523 | 211 | 38 | 145 | 103 | 22.9 |
| C 524 | 145 | 49 | 82 | 60 | 7.9 |
| C 525 | 131 | 54 | 69 | 49 | 32.6 |
| C 526 | 199 | 49 | 129 | 68 | 7.9 |
| C 529 | 181 | 57 | 108 | 74 | 7.9 |
| C 531 | 200 | 46 | 131 | 109 | 10.2 |
| C 534 | 214 | 55 | 135 | 81 | 7.9 |
| C 536 | 220 | 66 | 136 | 37 | 13.5 |
| C 547 | 165 | 48 | 100 | 75 | 57.7 |

**Supplementary Table S6: List of primers used for Quantitative Real-Time PCR.**

| **Gene** | **Primer Sequence** |
| --- | --- |
| hACAT1 | fw: 5´- ATG CCA GTA CAC TGA ATG ATG G -3´  rev: 5´- GAT GCA GCA TAT ACA GGA GCA A-3´ |
| hRPL30 | fw: 5´- CTG GTG TCC ATC ACT ACA GTG G -3´  rev: 5´- CCA GTC TGT TCT GGC ATG CTT C-3´ |
| hABCA1 | fw: 5´- AGT TAG GAA ACC TGC TGC CC - 3´  rev: 5´ - GTA GAT GAT GCC CCC ACA GG - 3´ |
| hHMGCR | fw: 5´- ATG CCC ATC CCT GTT GGA GT- 3´  rev: 5´ - CCA CGA GTC ATC CCA TCT GC - 3´ |
| hHPRT | fw: 5´- TGA CAC TGG CAA AAC AAT GCA- 3´  rev: 5´- GGT CCT TTT CAC CAG CAA GCT- 3´ |

**Supplementary figure legends:
Supplementary figure 1. Tumor classification of LUAD patients in dependence of the BMI.**
**a)** BMI of the LUAD patients in dependence of the grading (Mean: G1: 29.8; G2: 27.4; G3: 26.7; control group: 23.6). **b)** BMI of the LUAD patients in dependence of the TNM classification (Mean: Stage I: 28.4; Stage II: 26.8; Stage III: 25.8; Stage IV: 23.9; control group: 23.6). **c)** Direct correlation between the BMI and the glucose values before surgery of all LUAD patients (G1+G2+G3) in dependence of the grading (n=59, p=0.9531, R^2^=6.133e-005).

**Supplementary figure 2. Tumor classification of LUAD patients in dependence of triglycerides and HDL cholesterol.**
**a)** Triglyceride values before surgery in dependence of the grading (Mean: G1: 146.2; G2: 121.4; G3: 125; control group: 87.2). **b)** Triglyceride values before surgery in dependence of the TNM classification (Mean: Stage I: 143.4; Stage II: 136; Stage III: 102.8; Stage IV: 129.6; control group: 87.2). **c)** HDL cholesterol values before surgery in dependence of the grading (Mean: G1: 48.8; G2: 41.1; G3: 48; control group: 54.3). **d)** HDL cholesterol values before surgery in dependence of the TNM classification (Mean: Stage I: 45; Stage II: 42.9; Stage III: 48.4; Stage IV: 37; Control group: 54.2). **e)** LDL cholesterol values before surgery in dependence of the TNM classification (Mean: Stage I: 113.8; Stage II: 108.2; III: 98.3; Stage IV: 100.8)

**Supplementary figure 3. Correlation between the triglyceride values before surgery and the tumor diameter in dependence of the grading/the TNM classification.**
**a)** Direct correlation between the triglyceride values before surgery and the tumor diameter of all LUAD patients (G1+G2+G3) in dependence of the grading (n=65, p=0.2571, R^2^=0.02034). **b)** Direct correlation between the triglyceride values before surgery and the tumor diameter of all LUAD patients (Stage I+II+III+IV) in dependence of the TNM classification (n=67, p=0.2391, R^2^=0.02125). c) Direct correlation between the Lipoprotein a values before surgery and the tumor diameter of all LUAD patients (G1+G2+G3) (n=65, p=0.9332, R^2^= 0.0001221)

**Supplementary figure 4. Pre-surgical HDL cholesterol and lipoprotein a values of LUAD patients with Stage IV inversely correlate with the BMI.**
**a)** Direct correlation between the HDL cholesterol values before surgery and the BMI of all LUAD patients with Stage IV in dependence of the TNM classification (n=4, p=0.0294, R^2^=0.9420). **b)** Direct correlation between the Lipoprotein a values before surgery and the BMI of all LUAD patients with Stage IV in dependence of the TNM classification (n=4, p=0.0196, R^2^=0.9611).

**Supplementary figure 5. Expression of cholesterol biosynthesis-pathway related genes in tumoral and control lung tissue in dependence of the TNM classification**.
**a)** qPCR analysis of ACAT1/RPL30 mRNA expression level subdivided into TNM classification (Stage I: n(CTR)= 16, n(TU)= 15; Stage II: n (CTR/TU)= 5; Stage III: n(CTR)= 8, n(TU) =7; Stage IV: n (CTR/TU)= 3). **b)** qPCR analysis of HMGCR/RPL30 mRNA expression level subdivided into TNM classification (Stage I: n(CTR/TU)= 16; Stage II: n (CTR/TU)= 5; Stage III: n(CTR)= 9, n(TU) =8; Stage IV: n (CTR/TU)= 3). **c)** qPCR analysis of ABCA1/HPRT mRNA expression level subdivided into TNM classification (Stage I: n(CTR)= 16, n(TU)= 15; Stage II: n (CTR/TU)= 5; Stage III: n(CTR)= 8, n(TU) =7; Stage IV: n (CTR/TU)= 3).
Statistical analysis was performed using the Kruskal Wallis test.
