# Supplementary figures and images for: Contribution of serum lipids and cholesterol cellular metabolism in lung cancer development and progression
Source: Sci Rep. 2023 Apr 6;13:5662. doi: 10.1038/s41598-023-31575-y (PMC10079859; doi:10.1038/s41598-023-31575-y)

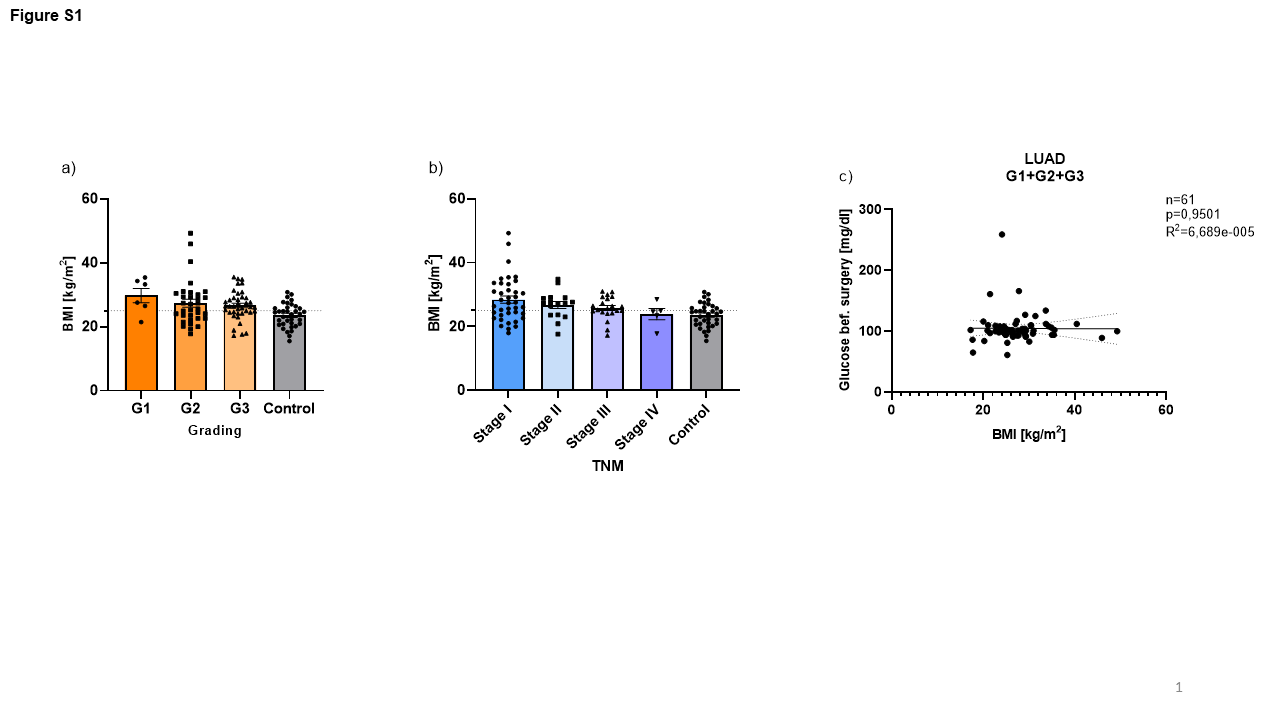

Supplement: Supplementary file 2 — Supplementary Figure 1. [file 41598_2023_31575_MOESM2_ESM.tif]

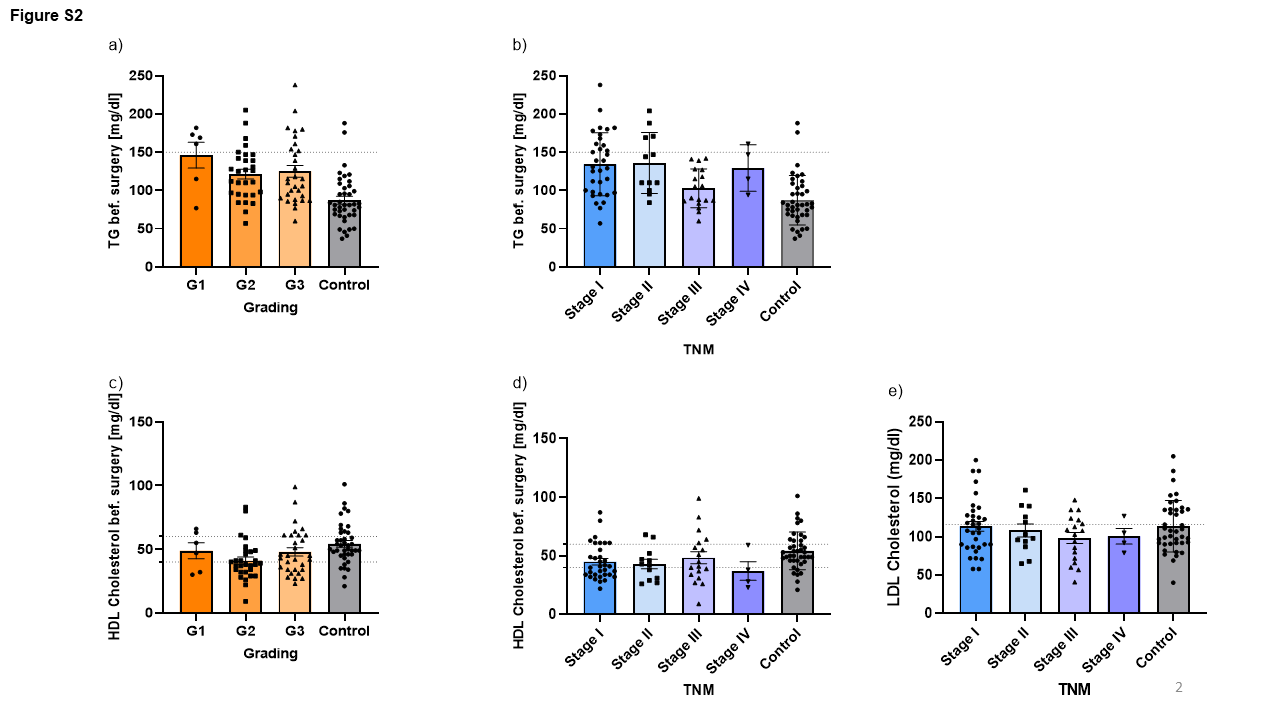

Supplement: Supplementary file 3 — Supplementary Figure 2. [file 41598_2023_31575_MOESM3_ESM.tif]

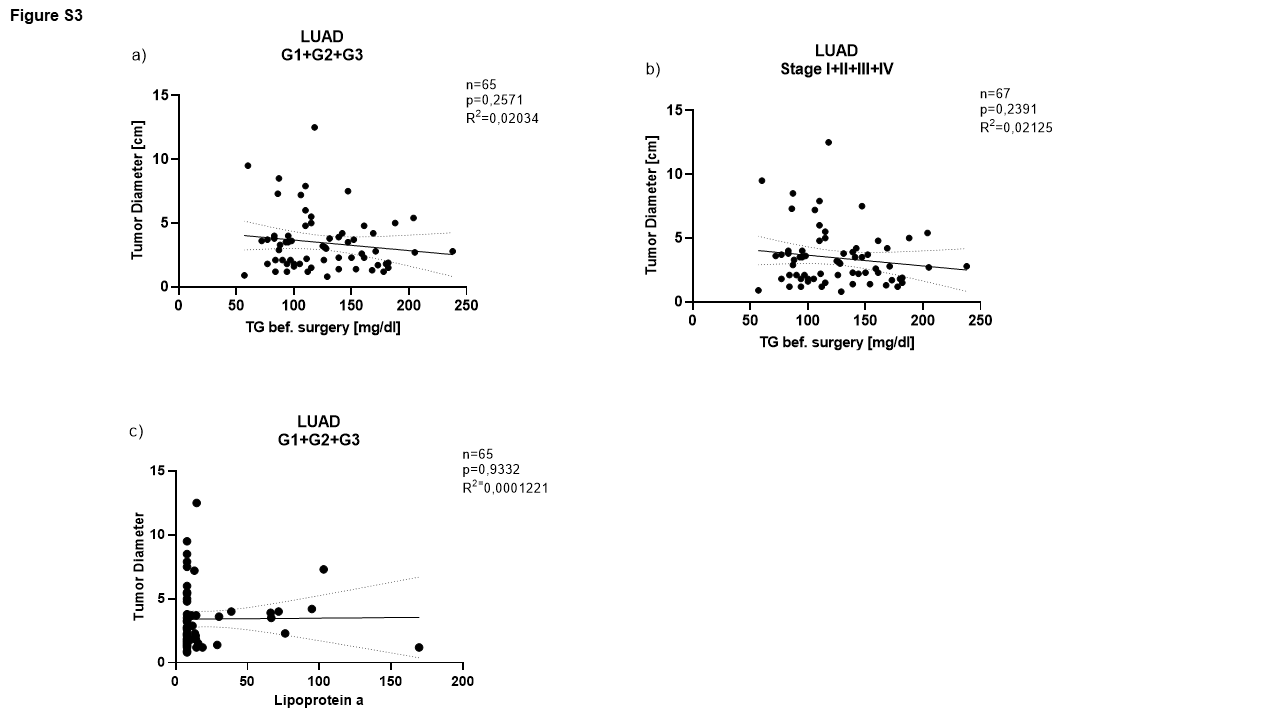

Supplement: Supplementary file 4 — Supplementary Figure 3. [file 41598_2023_31575_MOESM4_ESM.tif]

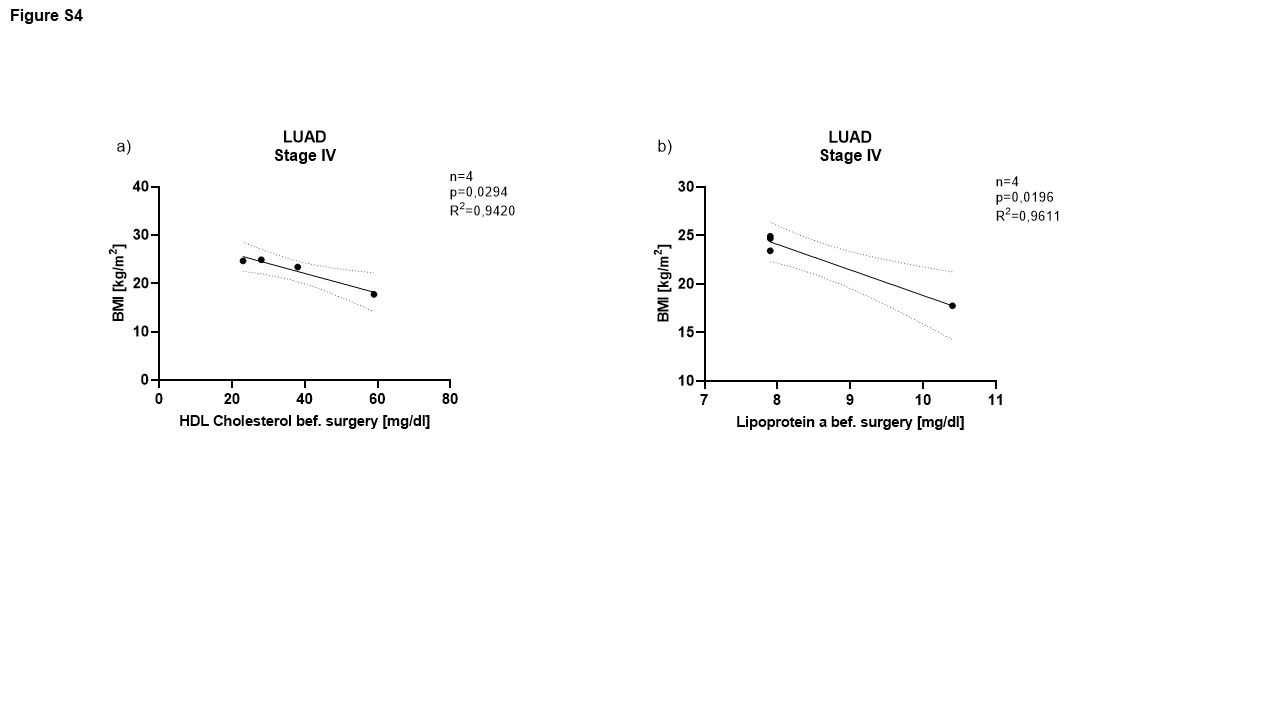

Supplement: Supplementary file 5 — Supplementary Figure 4. [file 41598_2023_31575_MOESM5_ESM.tif]

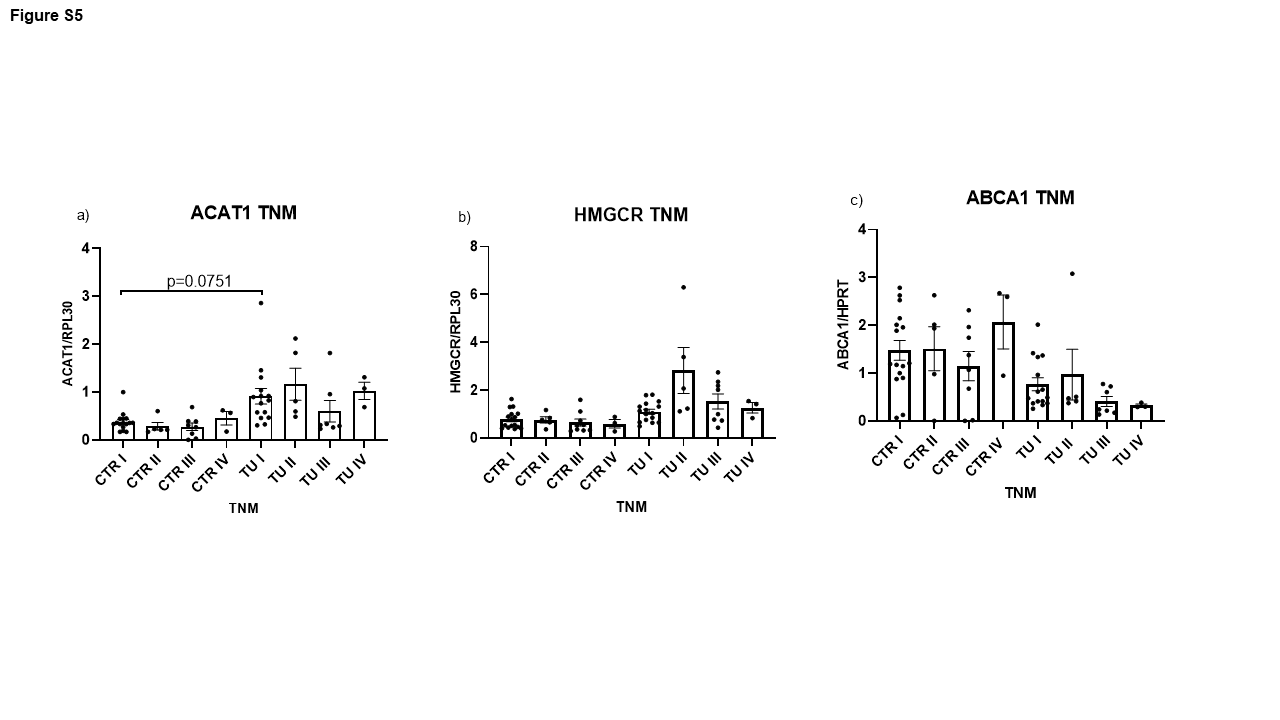

Supplement: Supplementary file 6 — Supplementary Figure 5. [file 41598_2023_31575_MOESM6_ESM.tif]
